# Oncolytic DNX-2401 virotherapy plus pembrolizumab in recurrent glioblastoma: a phase 1/2 trial

**DOI:** 10.1038/s41591-023-02347-y

**Published:** 2023-05-15

**Authors:** Farshad Nassiri, Vikas Patil, Leeor S. Yefet, Olivia Singh, Jeff Liu, Rachel M. A. Dang, Takafumi N. Yamaguchi, Mariza Daras, Timothy F. Cloughesy, Howard Colman, Priya U. Kumthekar, Clark C. Chen, Robert Aiken, Morris D. Groves, Shirley S. Ong, Rohan Ramakrishna, Michael A. Vogelbaum, Simon Khagi, Thomas Kaley, Jason M. Melear, David M. Peereboom, Analiz Rodriguez, Maxim Yankelevich, Suresh G. Nair, Vinay K. Puduvalli, Kenneth Aldape, Andrew Gao, Álvaro López-Janeiro, Carlos E. de Andrea, Marta M. Alonso, Paul Boutros, Joan Robbins, Warren P. Mason, Adam M. Sonabend, Roger Stupp, Juan Fueyo, Candelaria Gomez-Manzano, Frederick F. Lang, Gelareh Zadeh

**Affiliations:** 1 Division of Neurosurgery, University of Toronto, Toronto, Ontario Canada; 2 Princess Margaret Cancer Center, University Health Network, Toronto, Ontario Canada; 3 Department of Human Genetics, University of California Los Angeles, Los Angeles, CA USA; 4 Division of Neuro-oncology, University of California San Francisco, San Francisco, CA USA; 5 UCLA Neuro-Oncology Program, David Geffen School of Medicine, University of California Los Angeles, Los Angeles, CA USA; 6 Huntsman Cancer Institute and Department of Neurosurgery, University of Utah, Salt Lake City, UT USA; 7 Department of Neurology, Division of Neuro-Oncology, Northwestern University Feinberg School of Medicine, Chicago, IL USA; 8 Department of Neurosurgery, University of Minnesota, Minneapolis, MI USA; 9 Rutgers Cancer Institute of New Jersey, Rutgers University, New Brunswick, NJ USA; 10 Department of Neurology, Texas Oncology, Austin, TX USA; 11 Division of Neuro-Oncology, Department of Neurology, the Ohio State University Wexner Medical Center, Columbus, Ohio USA; 12 Department of Neurological Surgery, Weill Cornell Medical College, New York Presbyterian Hospital, New York, NY USA; 13 Department of Neuro-Oncology, Neuro-Oncology Program, Moffitt Cancer Center, Tampa, FL USA; 14 Division of Medical Oncology, University of North Carolina at Chapel Hill, Chapel Hill, NC USA; 15 Department of Neurology, Memorial Sloan Kettering Cancer Center, New York, NY USA; 16 Department of Internal Medicine, Baylor University Medical Center, Dallas, TX USA; 17 The Rose Ella Burkhardt Brain Tumor and Neuro-Oncology Center, Cleveland Clinic, Cleveland, OH USA; 18 Department of Neurosurgery, University of Arkansas for Medical Sciences, Little Rock, AK USA; 19 Department of Pediatrics, University of Michigan, Ann Arbor Beaumont Children’s Hospital, Royal Oak, MI USA; 20 Lehigh Valley Topper Cancer Institute, Allentown, PA USA; 21 Department of Neuro-Oncology, The University of Texas MD Anderson Cancer Center, Houston, TX USA; 22 Laboratory of Pathology, National Cancer Institute, Bethesda, MD USA; 23 Department of Laboratory Medicine and Pathobiology, University Health Network, Toronto, Ontario Canada; 24 Department of Pathology, Clínica Universidad de Navarra, Pamplona, Spain; 25 Navarra Institute for Health Research (IdISNA), Pamplona, Spain; 26 Department of Pediatrics, Clínica Universidad de Navarra, Pamplona, Spain; 27 Program of Solid Tumors, Center for the Applied Medical Research (CIMA), Pamplona, Spain; 28 DNATrix Inc., Carlsbad, CA USA; 29 Department of Neurological Surgery, Feinberg School of Medicine, Northwestern University, Chicago, IL USA; 30 Northwestern Medicine Malnati Brain Tumor Institute of the Lurie Comprehensive Cancer Center, Feinberg School of Medicine, Northwestern University, Chicago, IL USA; 31 Department of Medicine, Division of Hematology/Oncology, Feinberg School of Medicine, Northwestern University, Chicago, IL USA; 32 Department of Neurology, Feinberg School of Medicine, Northwestern University, Chicago, IL USA; 33 Department of Neurosurgery, The University of Texas MD Anderson Cancer Center, Houston, TX USA; 34 Department of Surgery, University of Toronto, Toronto, Ontario Canada

**Keywords:** CNS cancer, Phase II trials

## Abstract

Immune-mediated anti-tumoral responses, elicited by oncolytic viruses and augmented with checkpoint inhibition, may be an effective treatment approach for glioblastoma. Here in this multicenter phase 1/2 study we evaluated the combination of intratumoral delivery of oncolytic virus DNX-2401 followed by intravenous anti-PD-1 antibody pembrolizumab in recurrent glioblastoma, first in a dose-escalation and then in a dose-expansion phase, in 49 patients. The primary endpoints were overall safety and objective response rate. The primary safety endpoint was met, whereas the primary efficacy endpoint was not met. There were no dose-limiting toxicities, and full dose combined treatment was well tolerated. The objective response rate was 10.4% (90% confidence interval (CI) 4.2–20.7%), which was not statistically greater than the prespecified control rate of 5%. The secondary endpoint of overall survival at 12 months was 52.7% (95% CI 40.1–69.2%), which was statistically greater than the prespecified control rate of 20%. Median overall survival was 12.5 months (10.7–13.5 months). Objective responses led to longer survival (hazard ratio 0.20, 95% CI 0.05–0.87). A total of 56.2% (95% CI 41.1–70.5%) of patients had a clinical benefit defined as stable disease or better. Three patients completed treatment with durable responses and remain alive at 45, 48 and 60 months. Exploratory mutational, gene-expression and immunophenotypic analyses revealed that the balance between immune cell infiltration and expression of checkpoint inhibitors may potentially inform on response to treatment and mechanisms of resistance. Overall, the combination of intratumoral DNX-2401 followed by pembrolizumab was safe with notable survival benefit in select patients (ClinicalTrials.gov registration: NCT02798406).

## Abstract (web summary)

A safety and efficacy trial of a single intratumoral dose of the oncolytic adenovirus DNX-2401 followed by intravenous anti-PD-1 pembrolizumab in patients with recurrent glioblastoma shows an encouraging clinical benefit rate and 12 months overall survival.

## Main

Glioblastoma is the most common and lethal adult primary brain tumor. The standard of care treatment for newly diagnosed patients includes surgical resection followed by concomitant chemoradiotherapy and adjuvant temozolomide^1^. Despite maximal multimodal therapy, patients invariably experience recurrence of their disease 7 months after diagnosis, on average^1^. Unfortunately, treatment options at recurrence are scarce. Existing salvage therapies have very limited efficacy, with median survival being in the range of only 6–8 months after tumor progression^2^. Effective treatments for recurrent disease are urgently needed.

While immune checkpoint blockade by anti-PD1 or anti-PD-L1 antibodies have improved outcomes with objective responses in a variety of other cancers, including those in the brain such as metastatic melanoma^3^, they have had limited efficacy as monotherapy for recurrent glioblastoma where the microenvironment is innately immunosuppressive (that is, immunologically ‘cold’)^4,5^. Oncolytic viruses are capable of reconditioning the tumor microenvironment toward a ‘hot’ phenotype, providing rationale for combinatorial therapy with checkpoint inhibitors, which has been shown to improve outcomes in other cancers^6,7^.

DNX-2401 (tasadenoturev; Delta-24-RGD) is a conditionally replicative oncolytic adenovirus engineered to treat high-grade malignant gliomas^8,9^. The virus contains two stable genetic changes in the adenovirus dsDNA genome that cause it to selectively and efficiently replicate in cancerous cells. A dose-escalation phase 1 study demonstrated that stereotactic delivery of DNX-2401 into patients with high-grade gliomas was safe and induced cell death initially by direct oncolysis and subsequently by antitumor response from infiltrated immune cells, with durable responses after a single intratumoral dose^10^. In this Article, we report the results of CAPTIVE (2401BT-002P; KEYNOTE-192; NCT02798406), a two-part, phase 1/2, multicenter, open-label clinical trial of combined intratumoral injection of DNX-2401 with systemic pembrolizumab for patients with recurrent glioblastoma. This is the first in-human investigation of combined oncolytic virus with immune checkpoint blockade for recurrent glioblastoma.

## Results

### Patient demographics and baseline characteristics

**Fig. 1:**
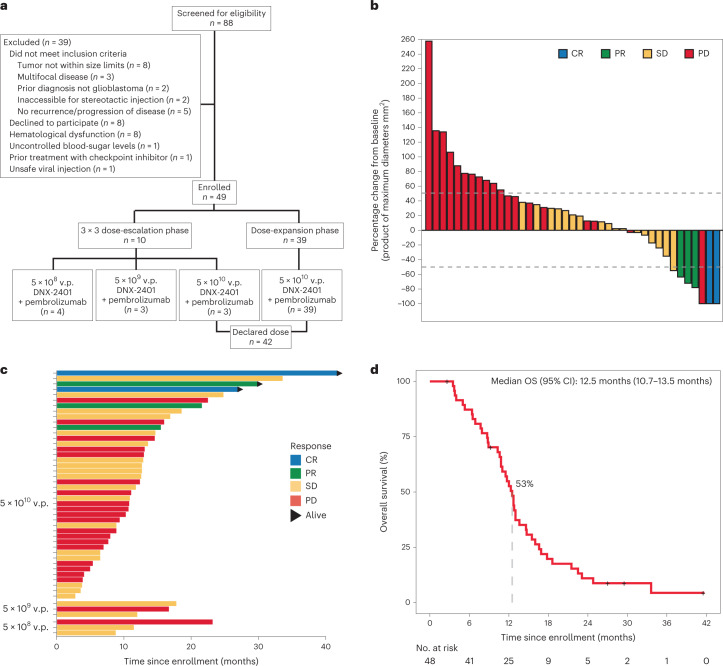
Survival and response to treatment. **a**, Patient flow in trial. **b**, Waterfall plot that displays the maximal change in tumor size for all patients who received full-dose DNX-2401 treatment (*n* = 42). Bars represent the maximal tumor change from baseline on the basis of contrast-enhanced MRI. Bars are colored according to responses classified according to mRANO criteria. **c**, Survival for each patient by DNX-2401 dose. The bar colors show the response to treatment according to the mRANO criteria. Arrows indicate that the patient remains alive. **d**, Overall survival for the intent to treat population. Crosses denote censored data.

A total of 49 patients from 13 of the 15 participating institutions were enrolled between 28 September 2016 and 17 January 2019 (Fig. 1a). The demographic and baseline clinical characteristics of all patients enrolled are reported in Table 1. The median age of patients was 53 years, and 41% were women. The majority of patients (80%) presented after first recurrence, and 18% of patients were using steroids at baseline. All patients had histopathological diagnosis of glioblastomas, except one patient enrolled with gliosarcoma (2%). Most patients (90%, *N* = 44) had reported IDH1 wild-type tumors, four (8%) had IDH1 mutant tumors and IDH1 mutation status was not known for one patient. All patients had received prior treatment with temozolomide and radiotherapy, six (12%) patients had prior bevacizumab treatment and five (10%) had prior treatment with a tumor-treating fields device.

**Table 1:** Patient demographics and baseline characteristics

Characteristic	Patients (*N* = 49), no. (%)
Median age (range), years	53 (26–73)
Sex	
Female	20 (41%)
Male	29 (59%)
Diagnosis at enrollment	
Glioblastoma	48 (98%)
Gliosarcoma	1 (2%)
Karnofsky Performance Score	
100	10 (20%)
90	28 (57%)
80	6 (12%)
70	5 (10%)
Recurrences before treatment	
1	39 (80%)
2	10 (20%)
Baseline tumor size	
Median maximum diameter (range), mm	28.5 (11.0–48.2)
Median tumor area (range), mm^2^	597.2 (110.0–1599.8)
*IDH1* R132 status	
Mutant	4 (8%)
Wild type	44 (90%)
Unknown	1 (2%)
*MGMT* gene promoter methylation status	
Methylated	14 (29%)
Unmethylated	28 (57%)
Unknown	7 (14%)
Prior therapies	
Surgical resection	44 (90%)
Radiotherapy	49 (100%)
Temozolomide	49 (100%)
Bevacizumab	6 (12%)
Tumor treating fields	5 (10%)
Baseline dexamethasone use ≥1.5 mg per day	
Yes	9 (18%)
No	40 (82%)

### Safety

Forty-eight of 49 (98%) patients were treated with one dose of DNX-2401 after a standard biopsy, which was then followed by pembrolizumab starting 7 days later. One patient enrolled in the first dose cohort received 5 × 10^8^ viral particles (v.p.) DNX-2401 but did not start pembrolizumab due to delirium, which was attributed by the investigators to anesthesia used during biopsy, unrelated to treatment. This patient was included in the safety analysis set only, per protocol. There were no dose-limiting toxicities observed, and the maximal dose tested (5 × 10^10^ v.p. DNX-2401) was selected as the declared dose for the dose-expansion phase. In total, across both dose-escalation and dose-expansion phases, patients were treated with 5 × 10^8^ (*n* = 4), 5 × 10^9^ (*n* = 3) and 5 × 10^10^ v.p. DNX-2401 (*n* = 42). The median duration of exposure to treatment with DNX-2401 and pembrolizumab was 153 days (range 21–753 days), including three patients (6%) who completed the full 2 year course of pembrolizumab therapy.

An overview of adverse events (AEs) in the study is summarized in Extended Data Tables 1 and 2 and Supplementary Table 1. Overall, DNX-2401 in combination with pembrolizumab was generally well tolerated and AEs were primarily as expected for patients with recurrent glioblastoma, with the majority of these being grade 3 or lower events. There were no AEs related to adenoviral infection. There were no deaths related to AEs that were related to treatment. One patient died approximately 7 months after initiating treatment due to hyperosmolar hyperglycemic nonketotic acidosis, which was considered unrelated to treatment.

**Extended Data Fig. 1:**
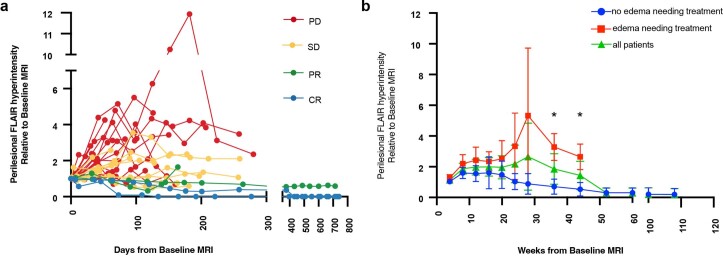
Longitudinal volumetric changes in perilesional edema. **a**, changes in perilesional FLAIR signal hyperintensity at each study MRI time point for individual patients. Patients are colored according to their mRANO responses. **b**, changes in perilesional FLAIR signal hyperintensity at each study MRI time point relative to baseline MRI. Data presented are mean +/− 95%CI. Patients are stratified by whether they developed clinically relevant edema requiring medical treatment. n = 9 independent patients developing edema requiring medical treatmet. * denotes statistical significance by two tailed unpaired *t*-test, 36 weeks P = 0.004 and 44 weeks P = 0.02.

AEs that were considered to be related to treatment are summarized in Table 2. The majority of these events were grade 1 or 2 events, with the most common being brain edema (37%), headache (31%) and fatigue (29%). Longitudinal volumetric changes of perilesional edema are shown in Extended Data Fig. 1. We found that patients with and without symptomatic edema both had increases in volumetric measurements of perilesional edema from 8 weeks to 20 weeks after treatment. Patients who did not develop symptomatic edema begin to have a decrease in volume of perilesional edema after 20 weeks, whereas those who develop symptomatic edema continue to have increases in volume of perilesional edema after 20 weeks. Treatment-related serious AEs that were noted in more than one patient included brain edema (16%), dysphasia (6%) and hemiparesis (6%). Serious cerebral edema was managed with either short-course dexamethasone (89%) and/or other concomitant supportive medications including bevacizumab (18%; Supplementary Table 2). Surgical intervention was not needed for serious cerebral edema in any patient. Pembrolizumab was interrupted or discontinued for four patients who had cerebral edema but resumed after resolution. One patient had grade 3 cerebral edema, somnolence and hemiparesis that started 23 days after initiation of treatment, leading to treatment discontinuation and resolution of the AE. A summary of serious AEs related to treatment is provided in Supplementary Table 3.

**Table 2:** Summary of AEs related to treatment

	Grade 1	Grade 2	Grade 3	Grade 4	Grade 5	Total
	*n* (%)	*n* (%)	*n* (%)	*n* (%)	*n* (%)	*n* (%)
AE related to treatment						
Overall frequency	6 (12%)	16 (33%)	11 (22%)	1 (2%)	0 (0%)	34 (69%)
Brain edema	2 (4%)	8 (16%)	7 (14%)	1 (2%)	0 (0%)	18 (37%)
Headache	4 (8%)	9 (18%)	2 (4%)	0 (0%)	0 (0%)	15 (31%)
Fatigue	7 (14%)	7 (14%)	0 (0%)	0 (0%)	0 (0%)	14 (29%)
Dysphasia	4 (8%)	4 (8%)	0 (0%)	0 (0%)	0 (0%)	8 (16%)
Hemiparesis	0 (0%)	2 (4%)	4 (8%)	0 (0%)	0 (0%)	6 (12%)
Pyrexia	5 (10%)	0 (0%)	0 (0%)	0 (0%)	0 (0%)	5 (10%)
Decreased appetite	3 (6%)	1 (2%)	0 (0%)	0 (0%)	0 (0%)	4 (8%)
Myalgia	2 (4%)	2(4%)	0 (0%)	0 (0%)	0 (0%)	4 (8%)
Nausea	2 (4%)	2(4%)	0 (0%)	0 (0%)	0 (0%)	4 (8%)
SAE related to treatment						
Overall frequency	0 (0%)	4 (8%)	9 (18%)	1 (2%)	0 (0%)	14 (29%)
Brain/vasogenic edema	0 (0%)	1 (2%)	6 (12%)	1 (2%)	0 (0%)	8 (16%)
Dysphasia	0 (0%)	3 (6%)	0 (0%)	0 (0%)	0 (0%)	3 (6%)
Hemiparesis	0 (0%)	0 (0%)	3 (6%)	0 (0%)	0 (0%)	3 (6%)

AE denotes adverse event. SAE denotes serious adverse event. Shown are AEs and SAEs with greater than 5% frequency. Overall frequency refers to patients reporting at least one treatment related AE. Each patient is included once using the highest-grade event. Events were graded according to National Cancer Institute-Common Terminology Criteria for Adverse Events, version 4.03.

### Efficacy

The efficacy and survival endpoints are summarized in Table 3. According to modified Response Assessment in Neuro-Oncology (mRANO) criteria, two patients had a complete response and three patients had a partial response (Fig. 1b,c) yielding an objective response rate of 10.4% (90% CI 4.2–20.7%) in the intent-to-treat population and 11.9% (90% CI 4.8–23.4%) for patients treated with the declared dose of DNX-2401, which was numerically greater than prespecified historical rate of 5% but did not meet statistical endpoint. One additional patient of interest had a complete response at the lesion where DNX-2401 was delivered approximately 8 months after treatment; however, a new lesion at a distant site was evident at the same assessment and the patient was therefore classified to have progressive disease. The median time to response was 3.0 months (range 1.9–17.4 months), and median duration of response was 9.4 months (range 1.8–33.7 months) in patients who showed an objective response. An additional 22 patients in the intent-to-treat population and 18 patients in the declared dose population had stable disease lasting longer than 28 days, which resulted in a clinical benefit rate of 56.2% (95% CI 41.1–70.5%) and 54.8% (95% CI 38.7–70.2%), respectively. The median duration of clinical benefit was 3.7 months (range 1.7–37.7 months). A summary of therapies received after treatment and at or after disease progression is presented in Supplementary Table 4.

**Table 3:** Summary of efficacy endpoints

Response, %	Intent-to-treat population, *N* = 48	Declared dose cohort, *N* = 42
Objective response rate (90% CI; 95% CI)	10.4% (3.5–22.3%; 4.2–20.7%)	11.9% (4.8–23.4%; 3.9–25.6%)
CR	4.1% (0.5–14.2%; 0.7–12.5%)	4.8% (0.9–14.2%; 0.6–16.1%)
PR	6.3% (1.3–17.1%; 1.7–15.3%)	7.1% (1.9–17.4%; 1.5–19.4%)
SD (95% CI)	45.8% (31.3–60.8%)	42.9% (27.7–59.0%)
PD (95% CI)	43.8% (29.4–58.8%)	45.2% (29.8–61.3%)
Clinical benefit rate (95% CI)	56.2% (41.1–70.5%)	54.8% (38.7–70.2%)
**Survival**		
12 month overall survival (95% CI)	52.7% (40.1–69.2%)	53.1% (36.8–67.0%)
Overall survival (95% CI)	12.5 months (10.8–14.6 months)	12.5 months (10.2–13.0 months)

CR denotes complete response; PR denotes partial response; SD denotes stable disease; PD denotes progressive disease. The primary efficacy endpoint was objective response rate, and the two secondary efficacy endpoints were clinical benefit rate and 12 month overall survival.

Patients with objective responses did not universally harbor characteristics that are commonly described in prognostically favorable tumors (Table 4). All patients with objective responses had reported *IDH1* wild-type tumors by immunohistochemistry (IHC), and only two of them had had tumors with *MGMT* promoter hypermethylation. Additional targeted sequencing revealed that two patients with objective responses harbored mutations in either *IDH1* or *IDH2* at low allelic frequencies. Three of the patients with objective responses only had prior radiation and chemotherapy without prior resection of their tumor. The median tumor diameter was similar in patients with and without objective response (32.8 mm, 95% CI 25.2–46.6 mm versus 28.4 mm, 95% CI 24.8–30.8 mm; Supplementary Fig. 1).

**Table 4:** Baseline characteristics of patients with complete or partial responses per mRANO criteria

	2401013	2401039	2401045	2401047	2401019
Response to treatment	CR	PR	CR	PR	PR
Maximum reduction in tumor volume from baseline	100	72.2	100	63.6	78.1
PFS, months	41.6^a^	5.3	26.8^a^	3.7	26.9
Survival, months	41.6 (alive)	21.5	26.8 (alive)	15.4	29.7 (alive)
Age, years	26	50	27	39	51
Recurrences, no.	1	1	2	1	2
Prior resection	No	Total resection	Total resection	No	No
Baseline steroids	Yes	Yes	No	No	No
*IDH1* R132H mutation status (IHC)	Wild type^b^	Wild type	Wild type	Wild type	Wild type^c^
*MGMT* promoter methylation status	Unknown	Unmethylated	Methylated	Unknown	Methylated
Maximal tumor diameter (mm)	40.4	32.8	38	19	28
Pretreatment PD-L1 expression by IHC	Unknown	Negative	Unknown	Negative	Positive
Tumor mutational burden (nonsynonymous mutations per 10 megabases)	5.7	7.8	10.0	7.8	10.0

CR denotes complete response; PR denotes partial response.

^a^Patients remain without progression at last follow-up.

^b^IDH1 wild type, but IDH2 mutation detectable by sequencing at allelic frequency of 23%.

^c^IDH1 R132H wild type by IHC, but detectable by sequencing at allelic frequency of 13%.

**Fig. 2:**
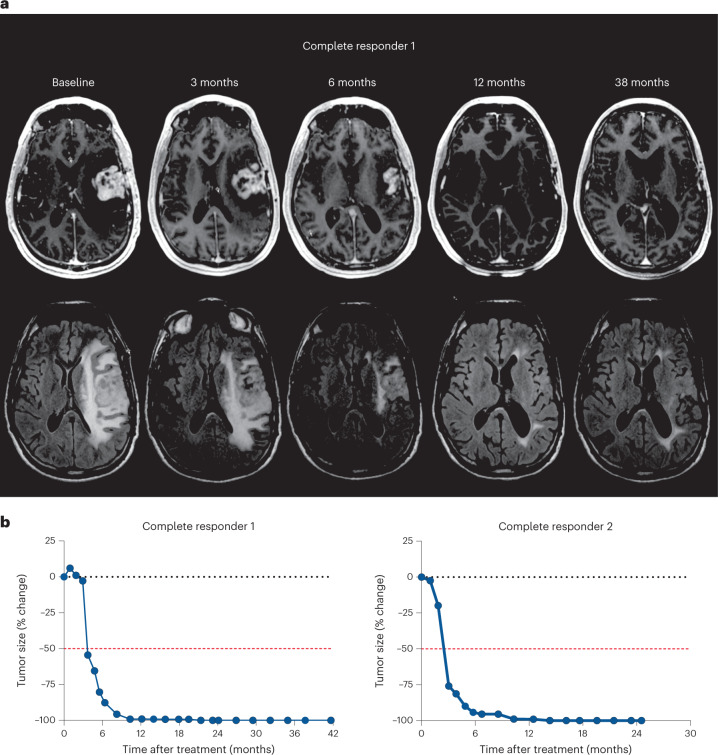
Complete responses to DNX-2401 and pembrolizumab. **a**, Axial T1-weighted MR (top row) and FLAIR images (bottom row) obtained at baseline, 3 months, 6 months, 12 months and 38 months after infusion of DNX-2401 for one complete responder. **b**, The change of tumor size over time in each patient with a complete response. Dotted black line represents no change relative to baseline. Dashed red line represents the threshold for response according to the mRANO criteria. Both patients showed response to treatment at 3 months after DNX-2401 infusion, with complete response by 15–18 months.

The two patients with complete response each had over 80% reduction in tumor volume approximately 6 months after treatment, which reached complete response criteria by 15–18 months after treatment (Fig. 2). These two patients completed 2 year treatment with pembrolizumab with durable responses and remain alive without evidence of disease progression.

### Survival analyses

**Extended Data Fig. 2:**
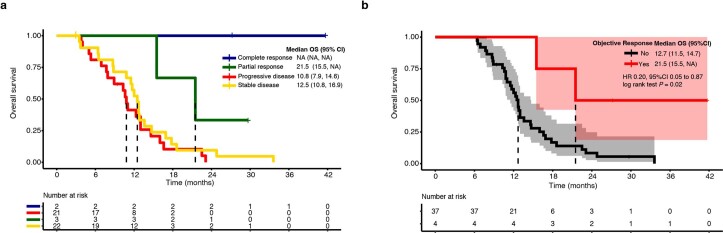
Survival of patients. **a**, Kaplan-Meier survival curve with patients stratified by response to treatment by mRANO. Crosses denote censored data. Dashes represent median overall survival for each group. **b**, Kaplan-Meier survival curve and associated 95%CI using landmark 6-months method with patients stratified according to objective response status. Crosses denote censored data. Dashed lines represent median overall survival for each group. Patients with objective responses (complete or partial response by mRANO) had statistically longer survival than those without objective responses (stable or progressive disease by mRANO, HR 0.20, 95%CI 0.05 to 0.87, log rank test P = 0.02).

The secondary efficacy endpoint of 12 month survival was met. The 12 month overall survival was 52.7% (95% CI 40.1–69.2%) in the intent-to-treat population and 53.1% (95% CI 36.8–67.0%) in patients who received the declared dose of DNX-2401 (Fig. 1d), and this was greater than the prespecified threshold of 20% from an approved treatment approach. The median overall survival was 12.5 months (10.7–13.5 months) in the intent-to-treat population and 12.5 months (95% CI 10.2–13.0 months) in declared dose population. Patients with objective responses had longer survival than patients without objective responses that was statistically significant (hazard ratio (HR) 0.20, 95% CI 0.05–0.87, *P* = 0.02; Extended Data Fig. 2). Three patients, all with objective responses (including the two patients with complete response), completed the prespecified pembrolizumab treatment and remain alive at the time this Article was written, beyond the study interval, at 45, 48 and 60 months. Moreover, one patient, with an *IDH1* wild-type and *MGMT* unmethylated tumor received a total of six doses of pembrolizumab with overall stable disease. This patient elected to discontinue participation in the study and remained alive over 34 months after initiation of treatment.

### Exploratory associations

We considered that concurrent use of medications may have impacted outcomes. Physicians were permitted to use low-dose bevacizumab or corticosteroids to address cerebral edema in this trial. Baseline corticosteroid use and corticosteroid use throughout the study were not statistically associated with outcomes, though use of corticosteroids throughout the study approached the threshold for statistical significance in some instances (Extended Data Table 3). Moreover, none of the patients with an objective response received bevacizumab during treatment.

**Extended Data Fig. 3:**
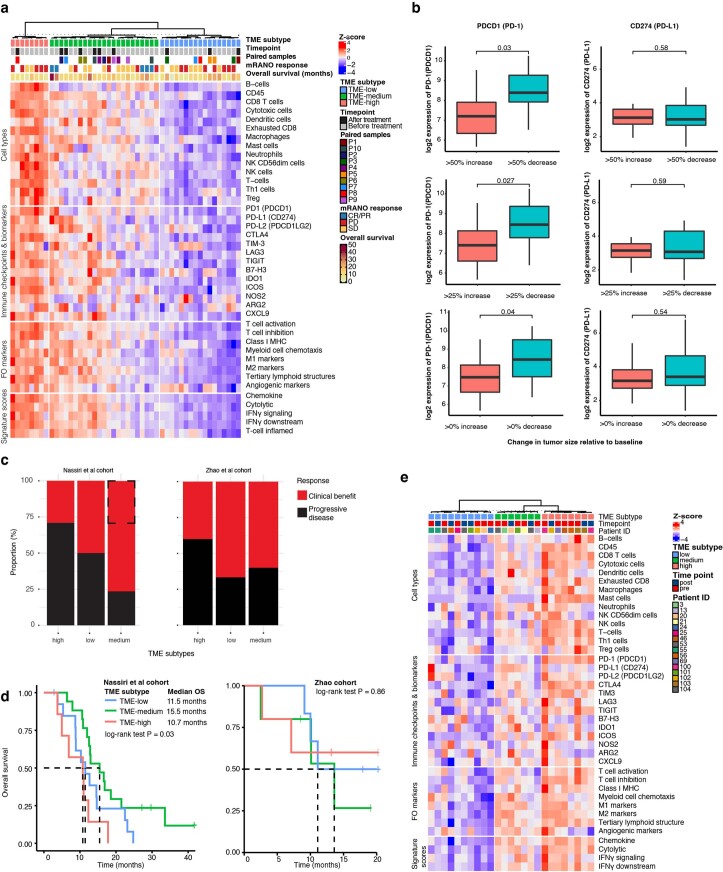
mRNA expression prior to treatment. **a**, Heatmap showing three subtypes of glioblastoma microenvironment in samples from this trial on the basis of enrichment for immune cell types using partition around medoids clustering. Scores for functional orientation markers, signature scores, and expression of immune checkpoints and biomarkers are overlayed on the heatmap. **b**, Distribution of expression of PD-1(PDCD-1) and PD-L1 (CD274) across different tumor size comparisons. n = 19 independent patients top row, n = 31 independent patients middle row, n = 38 independent patients bottom row. Central bars indicate medians, the box defines the upper and lower quartiles of the distribution, and whiskers define the 1.5× IQR. Statistical comparisons were performed using Welch’s two-sided *t*-test. **c**, Stacked barplot showing the rate of clinical benefit (stable disease or objective response) stratified by microenvironment subtypes in this study (left) and previously published cohort examining adjuvant PD-1 monotherapy in recurrent glioblastoma (right). Dashed box represents the proportion of objective responses by mRANO criteria in this study. **d**, Distribution of overall survival of patients in this trial (left) and previously published trial (right) stratified by immune microenivronment subtypes. e, Heatmap showing three subtypes of glioblastoma microenvironment on previously published cohort examining adjuvant PD-1 monotherapy in recurrent glioblastoma.

**Extended Data Fig. 4:**
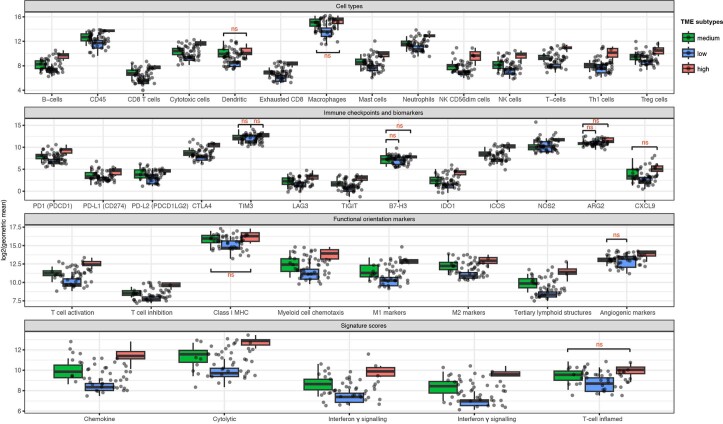
Immune cell types and signature markers. Distribution of scores for immune cell types (top row), immune checkpoint genes and biomarkers (second row), functional orientation markers (third row) and signature scores (last row). Shown are boxplots for each TME subtype. Dots denote individual values. N = 38 independent patient samples in total. Central bars indicate medians, the box defines the upper and lower quartiles of the distribution, and whiskers define the 1.5× IQR. All comparisons have p < 0.05 unless specifically indicated with ns (p > 0.05).

We also considered that variability in intrinsic patient and tumor factors might be associated with differences in outcomes of patients. To characterize potential biomarkers of treatment response, we obtained gene expression data on 38 patients with biopsy specimens available before treatment. We divided tumors from this study into three tumor microenvironment subtypes (TME^high^, TME^medium^ and TME^low^) on the basis of the degree of immune cell enrichment (Extended Data Fig. 3a), as recently described^11^. TME^high^ tumors had high scores for multiple different immune cells but also highly expressed multiple complementary suppressive immune checkpoints genes (Extended Data Fig. 4). By contrast, TME^low^ tumors had low immune cell scores with low expression of immune checkpoint genes. TME^medium^ tumors had intermediary immune cell scores and expression of *PDCD-1* (gene that encodes PD-1) but relatively low expression of other checkpoint proteins. We found that pre-treatment gene expression levels of *PDCD-1*, but not *CD274 (*gene that encodes PD-L1), was statistically significantly associated with reduction in tumor size (Extended Data Fig. 3b and Supplementary Fig. 2). All of the patients who had an objective response had TME^medium^ tumors before treatment (29.4%, 95% CI 10.3–55.6%, *P* = 0.012). Patients with TME^medium^ tumors were more likely to have clinical benefit from treatment (odds ratio (OR) 4.08, 95% CI 1.02–19.4, *P* = 0.036; Extended Data Fig. 3c), and also had statistically significantly longer survival in our cohort (HR 2.27, 95% CI 1.09–4.49, *P* = 0.027; Extended Data Fig. 3d). Patient samples from a prior trial investigating adjuvant anti-PD1 monotherapy in recurrent glioblastoma^12^ were also divisible into the same three TME subtypes, but associations between TME subtypes and outcomes were less clear in this population treated with monotherapy (Extended Data Fig. 3c,e).

**Extended Data Fig. 5:**
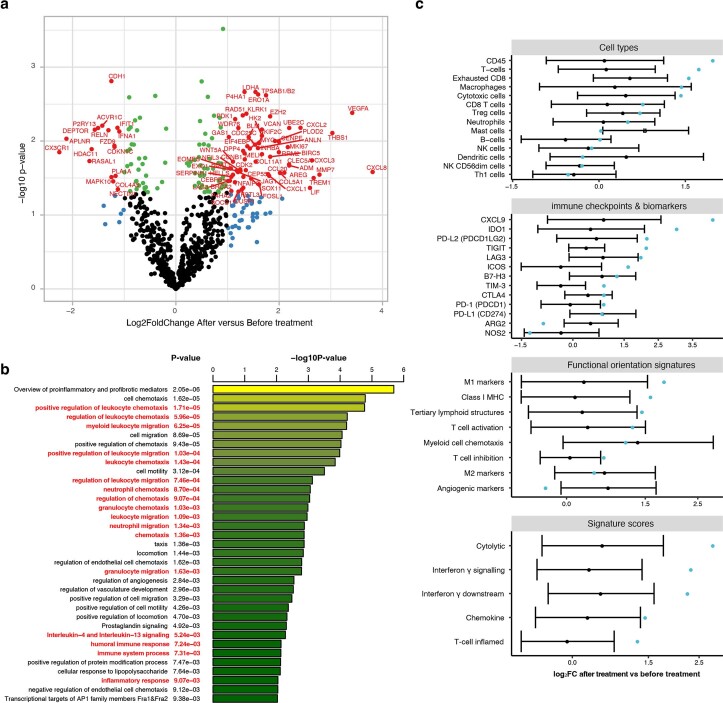
Comparison of gene mRNA expression at disease progression to baseline. **a**, Volcano plot showing the expression changes of individual genes after treatment compared to baseline. Genes that are differentially expressed (absolute Log_2_FC greater than 1 and P-value using two-sided Welch’s *t*-test less than 0.05) are colored red and labelled. Blue dots indicate genes with absolute Log_2_FC greater than 1. Green dots indicate genes with P value < 0.05**. b**, Barplot showing results of functional enrichment analysis of differentially expressed genes in (a). P-values generated using two-sided limma *t*-test. **c**, Forest plot summarizing differences in gene expression profiles and signatures for 9 patients who did not have objective response to treatment (shown in black) with expression data at both disease progression and baseline. Dot denotes average and bars represent 95%CI. Blue dot denotes one additional patient with objective partial response to treatment. FC denotes fold change.

Ten patients also had biopsy specimens at the time of disease progression after treatment allowing for a biological assessment of matched-pair tissues. Of these ten patients, one initially had a partial response to treatment before progression, while the other nine patients did not demonstrate objective responses (three patients with progressive disease as best response and six patients with initially stable disease as best response). Comparing gene expression profiles at disease progression after treatment to those at baseline before treatment revealed several differentially expressed genes (Extended Data Fig. 5a). Genes that were overexpressed in post-treatment specimens were highly enriched for pathways involved in immune system activation and regulation by functional enrichment analysis (Extended Data Fig. 5b). The patient with a partial response to treatment showed heightened immune activity after treatment relative to other patients, with the highest levels of interferon gamma and downstream signaling, infiltration of T cells, as well as the highest score for a T-cell inflamed microenvironment (Extended Data Fig. 5c)^13^. Moreover, the expression of several different immune checkpoint genes such as TIGIT (log_2_ fold change (FC) 1.77), LAG3 (log_2_FC 2.05) and CD276 (log_2_FC 2.06) were consistently increased in post-treatment samples, and this was highest for the patient with a partial response to treatment.

**Extended Data Fig. 6:**
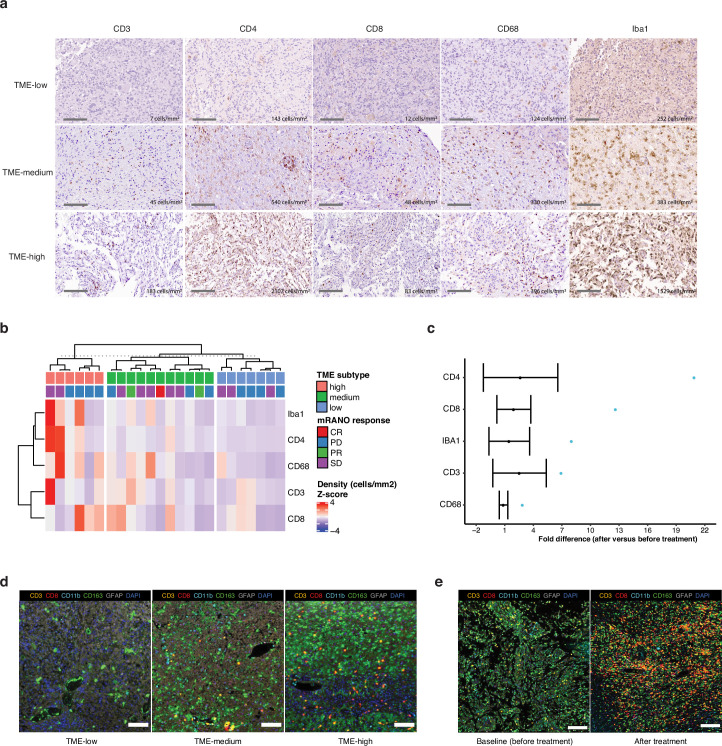
Immunophenotyping before and after treatment. **a**, Representative immunohistochemical stains for microglia (Iba1), macrophages (CD68), and lymphocytes (CD3, CD4, CD8) for patients with low, moderate, and high microenvironment subtypes in this trial. Scale bar = 100um. Experiments were completed once. **b**, Heatmap showing relative density (cells per mm^2^) of markers stratified by microenvironment subtype as determined by PAM clustering of immune cell type scores from gene expression deconvolution **c**, Forest plot summarizing fold differences in density of markers for 6 patients who did not have objective response to treatment (shown in black) with expression data at both disease progression and baseline. Dot denotes average and bars represent 95%CI. Blue dot denotes one additional patient with objective partial response to treatment. **d**, Representative immunofluorescence images of samples with different microenvironment subtypes as determined by gene expression analysis. Scale bar = 100um. Experiments were completed once. **e**, Immunofluoresence images of patient with objective response before (left) and after (right) treatment. Scale bar = 150um. Experiments were completed once.

We performed immunophenotypic characterization of tumors before and after treatment by blinded immunohistochemical and multiplex immunofluorescence analysis. Patients with TME^medium^ and TME^high^ tumors by gene expression subtyping also showed progressively greater density of immune cell infiltrates by IHC and immunofluorescence (Extended Data Fig. 6a–b,d). Comparing specimens before and after treatment, we found that increases in density of microglia (Iba1), macrophages (CD68) and lymphocytes (CD3, CD4 and CD8) after treatment were most evident in the patient who showed an objective response to treatment (Extended Data Fig. 6c,e).

Certain pathogenic mutations are potentially associated with prognosis and specific response to checkpoint inhibition in glioblastoma^14^. Clinically relevant molecular features were reported by investigators for tumor biopsies analyzed using various assays at each clinical site. Investigators reported MGMT status, IDH1/2 mutation and, for 42 of 49 subjects, pathogenic mutations. Targeted next-generation sequencing was also separately performed on available tumor biopsies on a subset of patients. A notable number of pathogenic mutations, including those in *TP53*, *NF1*, PTEN, MTOR and *RB1* were detected, as were a few mutations in *POLE* and *POLD1*. There was no clear association between these specific molecular features, including tumor mutational burden, on response to treatment (Table 4 and Supplementary Table 5).

**Extended Data Fig. 7:**
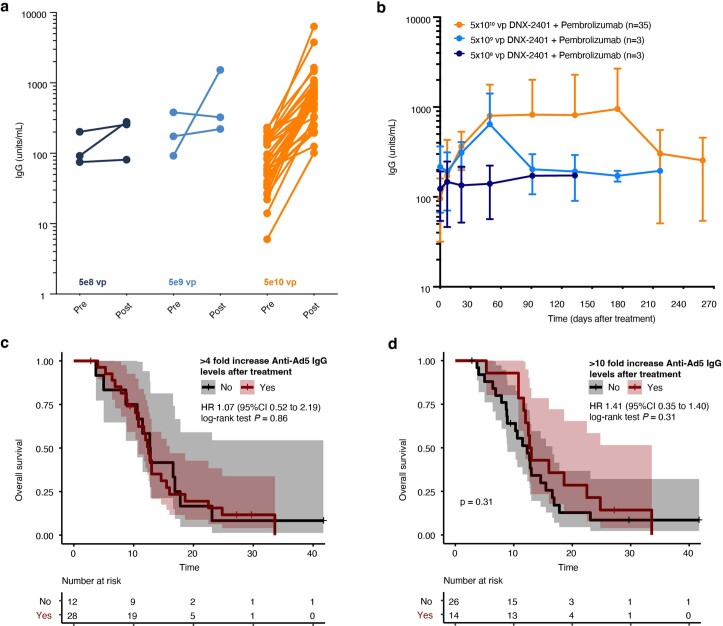
Anti-adenovirus antibody levels. **a**, Immunoglobulin G (IgG) levels before and after treatment across dose cohorts in the trial. **b**, IgG levels across dose cohorts in thet trial over time. Data shown are mean +/− SD. **c-d**, Shown are Kaplan-Meier survival curves with associated 95%CI. Patients stratified according to level of response in Anti-Ad5 IgG levels after treatment compared to baseline levels. + denote censored data. **c**, Shows survival using 4-fold threshold. **d**, Shows survival using 10-fold threshold.

Anti-adenovirus antibodies were measured by direct immunofluorescence assay in the serum of patients before treatment and throughout the course of the trial. All patients were seropositive for IgG antibodies against adenoviral hexon protein before treatment with DNX-2401, and in general, anti-adenovirus IgG levels increased within 2 months post treatment, with levels sustained longest in patients treated with 5 × 10^10^ v.p. DNX-2401, compared to lower doses (Extended Data Fig. 7a,b). We considered that variability in systemic immunogenic response to DNX-2401 might have impacted outcomes. The median overall survival of patients with and without a systemic immunogenic response to DNX-2401 delivery, which we defined as a greater than fourfold increase in baseline levels of anti-adenovirus antibodies, were similar (12.5 months, 95% CI 10.8–15.9 months versus 12.8 months, 95% CI 10.6 months to not reached). These findings were unchanged using more stringent thresholds of greater than tenfold increase in baseline levels of anti-adenovirus antibodies (12.9 months, 95% CI 12.0 months to not reached versus 12.3 months, 95% CI 8.9–16.6 months; Extended Data Fig. 7c,d)

## Discussion

Glioblastoma is a devastating disease, and recurrence of disease is inevitable after initial treatment with radiotherapy and concurrent and adjuvant temozolomide chemotherapy. At progression, treatment options are very limited and of marginal efficacy. Immune checkpoint blockade in other advanced solid cancers such as melanoma^15–17^ and non-small cell lung cancer^18,19^ has greatly improved outcomes. However, the innately immunologically cold microenvironment in glioblastomas has presumably rendered immune checkpoint blockade less effective for this disease^4,5^.

DNX-2401 (Delta-24-RGD) is a conditionally replicative oncolytic adenovirus with a 24 base pair deletion in the E1A gene that renders selective replication of the virus in malignant cells with defective retinoblastoma signaling. DNX-2401 also has an RGD peptide insertion into the fiber knob that allows the virus to anchor directly to integrins and improve the infectability of glioblastoma cells^9^. Preclinical studies of DNX-2401 in glioma mouse models showed promising antitumor immune activity as early as 1–2 weeks after delivery of a single dose of virus with potential for longer-term antigen-specific memory responses^9,20^. This led to the first in human trials of DNX-2401 for glioblastoma, where in addition to direct oncolytic effects, we showed that the delivery of the virus into tumors induced an immunogenic environment with increased T-cell infiltration and also altered the expression of checkpoint proteins^10^.

Treatment with oncolytic virus and immune checkpoint blockade combines the initial local effects of the oncolytic virus on the tumor microenvironment with the systemic effects of innate and adaptive immune responses from virus replication and PD-1 inhibition^7^. This combination has led to improved outcomes in other tumors, such as melanoma^6^, pointing to the possibility for therapeutic benefit of combination therapy in glioblastoma. Systematic screening of co-signaling molecules after DNX-2401 treatment in preclinical glioma models revealed significant increases in PD-1 expression that would prime the immune system for effective synergy with subsequent anti-PD-1 therapy^21^. Indeed, combination therapy of a single intratumoral dose of DNX-2401 followed by systemic pembrolizumab 1 week after viral treatment improved survival compared to monotherapy with either virus or pembrozliumab alone in glioma mouse models, providing rationale for further investigation in humans^21^.

Here we report the results of a two-part, phase 1/2, multicenter, open-label clinical trial evaluating the safety and efficacy of combined intratumoral delivery of DNX-2401 with systemic pembrolizumab for patients with recurrent glioblastoma treated at 13 institutions in North America. All centers used purpose-built cannulas to standardize the delivery of virus into the tumor, eliminating backflow and ensuring full administration of virus to the tumor. A total of 48 of 49 patients successfully received treatment with DNX-2401 and pembrolizumab.

We tested between 5 × 10^8^ to 5 × 10^10^ v.p. of DNX-2401 when delivered sequentially with pembrolizumab and found that the safety profile was consistent with prior studies reporting on oncolytic viruses or immunotherapies for brain tumors^3,10,22^. There were no dose-limiting toxicities in the dose-escalation phase of this study, and no deaths that were directly related to the treatment regimen. The most common serious AE reported was neurological symptoms related to increase in peritumoral inflammation (cerebral edema), which occurred in 16% of patients. We anticipated the possibility for treatment-induced cerebral edema when designing this study due to inflammatory responses observed in phase 1 study of DNX-2401 monotherapy^10^, and so we allowed for a short-course steroid or low-dose bevacizumab regimen to mitigate these effects. All serious cerebral edema events were resolved with anticipated medical measures, and surgical intervention to remove tumor due to tissue swelling was not necessary for any patient. We established the time course of edema development in this trial by serial volumetric analysis of changes in perilesional fluid-attenuated inversion recovery (FLAIR) signal on imaging. We found increases in volume of edema as early as 8 weeks after treatment that was sustained to 20 weeks, even in patients who did not become symptomatic with cerebral edema. These data can help inform on the expected time interval of cerebral edema for future trials of immunotherapy in recurrent glioblastoma. The nonneurologic toxicity profile in this study was otherwise comparable to those previously reported for pembrolizumab^5^.

In total, five patients had objective responses, with two patients showing durable complete responses >45 months and three patients remaining alive at the writing of this manuscript. The objective response rate was 10.4% (90% CI 4.2–20.7%). It is noteworthy that there was one additional patient who received the declared dose of DNX-2401 with complete response at the site of treatment; however, this patient developed a new lesion at a distant site resulting in a classification of progressive disease. This patient remained alive a total of 12.3 months after treatment. In the previous phase 1 trial evaluating DNX-2401 monotherapy in recurrent glioma, there was also one patient with a complete response who developed a distant nodule several years after treatment^10^. Pathological examination of the nodule after resection showed only necrosis and inflammation without evidence of tumor. Although the patient in this trial did not undergo resection for the new nodule, it is possible that the radiographic changes seen reflect a similar adaptive memory antitumor response that was observed in the original phase 1 trial of DNX-2401 monotherapy, and not progressive disease. Beyond this, prior reports of durable responses to immunotherapies have largely been limited to patients with favorable biological characteristics^23^. Patients with objective responses in this study had tumors that did not universally harbor the prognostically favorable mutation in *IDH1* and had both *MGMT* methylated and unmethylated tumors, representing the group of glioblastomas that desperately need efficacious therapies.

The median overall survival was 12.5 months (10.7–13.5 months) and overall survival at 12 months was 52.7% (95% CI 40.1–69.2%), which was greater than the prespecified threshold of 20% using approved treatment of tumor-treating fields by Novo-TTF^24^. The 12 month overall survival was 32% in patients treated with DNX-2401 alone^10^, while median overall survival was as 9.3 months and 9.8 months with DNX-2401 or PD-1 blockade alone in prior trials^5,10^. While the primary endpoint of objective response was not met, the secondary endpoint of 12 month survival, which is more clinically meaningful and reliable than response rate, was met and the survival of objective responders are encouraging, suggesting that tumor control led to improved survival. Although this trial was not designed to distinguish the effects of DNX-2401 versus pembrolizumab versus combination therapy, the notable survival data point to the potential of improved efficacy in combining oncolytic virus with checkpoint inhibition. As cross-trial comparisons have limitations, further focused comparative studies are needed.

While the use of bevacizumab may complicate response assessment in trials by inducing changes in contrast enhancement seen on imaging, none of the patients with objective responses received bevacizumab during the study. Moreover, we did not find that baseline corticosteroid use was associated with outcomes in our study, confirming the findings in a prior study evaluating neoadjuvant checkpoint blockade in recurrent glioblastoma^25^. This may be explained by the fact that patients using more than 4 mg per day of dexamethasone as baseline were excluded from both studies. Although associations of steroid use throughout this study and outcomes were not statistically significant, some comparisons approached the threshold for significance. Whether this association is reflective of symptom management in disease progression or a potential modulation of antitumor immune responses is unclear and warrants dedicated investigation in larger cohorts.

We obtained matched mutational data and gene expression data on tumor specimens from patients, where available. Three of the patients with objective responses (60%) had tumors with mutational burden (TMB) greater than 10 mutations Mb^−1^, while two patients with objective responses (40%) had tumors with TMB less than 10 mutations Mb^−1^. Although TMB is a known predictive biomarker of response to checkpoint inhibition in a range of advanced cancers, this relationship is more complex and has been less consistent in prior investigations in glioblastomas^26^. One of the major determinants linking TMB to response to checkpoint inhibition is alterations in mismatch repair proteins or polymerase E and D (*POLE* and *POLD*) genes^26^. None of the patients who showed objective responses had mutations in *POLE* or *POLD* genes. Although this suggests that the antitumor responses after combined oncolytic virus and checkpoint inhibition in glioblastomas may be less dependent on TMB than in other solid cancers, further investigation in much larger cohorts is warranted for definitive conclusions.

Using gene expression data, we found that objective responses exclusively occurred in patients with moderately inflamed microenvironment, and modest PD-1 expression (TME^medium^) before treatment (29.4%, 95% CI 10.3–55.6%). Clinical benefit rates and overall survival was also longer in TME^medium^ tumors in this trial. These findings are consistent with prior investigations and our own findings that show that adjuvant anti-PD1 inhibition as monotherapy does not seem to improve survival in TME^high^ tumors^11,25^. While TME^high^ tumors are enriched with immune cell infiltrates, they also highly express multiple different suppressive immune checkpoints leading to an exhaustive immune microenvironment by complementary mechanisms. TME^medium^ tumors are primed with a moderate degree of immune cells and express moderate levels of PD-1. DNX-2401 can induce further infiltration of cytotoxic T cells and expression of PD-1 in these tumors that can be further targeted with subsequent anti-PD-1 treatment without immunosuppression from alternative checkpoint proteins. We also obtained specimens on disease progression after treatment for ten patients in this trial. We found that the expression of several different immune checkpoints such as TIGIT, LAG3 and B7-H3 was elevated after treatment, pointing to the potential for using multiple parallel immune checkpoint inhibitors in TME^medium^ tumors that eventually develop disease progression. A similar approach could potentially be considered for TME^high^ tumors.

There are limitations to this study that require further investigation. First, this trial did not include a comparator cohort. Further trials to directly compare combination therapy to monotherapy are needed before considering large-scale randomized trials. Second, this trial evaluated a single dose of intratumoral oncolytic virus. Emerging data since the conception of this study have shown some potential benefit with multiple doses of oncolytic virus^27^. The safety of multiple doses of DNX-2401 with pembrolizumab needs further investigation given the local immune-stimulatory effects of treatment, if the logistical considerations to safely conduct such a trial can be addressed. Third, we did not find that variability in seroconversion, as measured by changes in anti-Ad5 IgG levels, impacted patient outcomes. While changes in anti-Ad5 IgG levels can be a surrogate for seroconversion, a potentially more definitive assessment of seroconversion would have benefited from quantification of neutralizing antibodies against human adenovirus^8^. Lastly, we identified biological correlates of outcome using gene expression, mutational data and immunophenotyping that can be leveraged to identify subsets of patients who might benefit most from treatment. It should be noted that these findings were exploratory, and future trials should consider maximizing collection of specimens before and after treatment to allow for even more comprehensive characterization of biological outcomes.

To our knowledge, the present study is the first to report on the combined direct delivery of oncolytic viral therapy and systemic checkpoint inhibition for any brain tumor. We identified a safe dose of DNX2401 combined with pembrolizumab with objective and durable responses, including two complete responses, and survival benefit for select patients across multiple institutions. These results are promising and particularly relevant in this population of patients who did not receive repeat resection of tumor and for whom efficacious and nontoxic treatments are entirely lacking. As well, we demonstrate the value that translational analyses and endpoints can add in advancing our understanding of the molecular mechanisms and biomarkers of response and/or resistance to treatment in clinical trial settings.

## Methods

### Patients

Adult patients with histologically confirmed glioblastoma or gliosarcoma, presenting with documented failure of previous surgical resection, chemotherapy and/or radiation at first or second recurrence, with a Karnofsky performance score of at least 70, were eligible. All patients were required to have a single contrast-enhancing tumor of at least 1 cm in two planes but no more than 4 cm in any single plane, as assessed by magnetic resonance imaging (MRI). Surgical resection must not have been possible or planned as part of the treatment for their presentation, and the tumor must have been accessible for stereotactic delivery of DNX-2401. Patients with multifocal or bilateral disease were excluded. The full inclusion and exclusion criteria are detailed in Supplementary Methods.

### Design

To evaluate the safety of combining DNX-2401 with pembrolizumab, we conducted an initial dose-escalation phase to determine a safe dose of DNX-2401 in combination with pembrolizumab and followed by a dose-expansion phase. All patients received a single dose of DNX-2401 by stereotactic injection at the time of standard tumor biopsy followed by 200 mg pembrolizumab infused intravenously at a dose of 200 mg over 30 min every 3 weeks starting 7 days after DNX-2401. Resection of tumors was not permitted. Treatment with pembrolizumab continued for up to 2 years, or until one of the following occurred: disease progression, unacceptable toxic effects or withdrawal of consent. Dose escalation evaluated 5 × 10^8^, 5 × 10^9^ and 5 × 10^10^ v.p. DNX-2401 in combination with standard dosing pembrolizumab in a 3 + 3 design.

All patients underwent a stereotactic biopsy to document the presence of tumor tissue before delivery of DNX-2401. Immediately after biopsy, a stereotactic-compatible neuro-ventricular cannula (Alcyone MEMS; ClearPoint SmartFlow) was inserted into the tumor to deliver the precise targeted dose of DNX-2401 via a single micro-tip at a rate of 0.9 ml h^−1^ over approximately 1 h. The cannula was left in place for 10 min after administration of virus to allow v.p. to diffuse without backflow before removal.

### Assessments

Patients were continuously monitored throughout the study for safety as outlined in the schedule of assessments in the study Protocol. AEs and serious AEs were graded according to National Cancer Institute-Common Terminology Criteria for Adverse Events, version 4.03, and their relationship to treatment administered was assessed. For the dose-escalation phase, the dose-limiting toxicity (DLT) window of observation was the first 21 days after initial pembrolizumab infusion. The occurrence of any of the following toxicities is considered a DLT, if judged by the Investigator to be possibly, probably or definitely related to administration of DNX-2401 and pembrolizumab (and not to the administration procedure):

1. Grade 4 nonhematologic toxicity (not laboratory)
2. Grade 4 hematologic toxicity lasting ≥7 days
3. Grade 3 nonhematologic toxicity (not laboratory) lasting >3 days despite optimal supportive care
4. Any Grade 3 or Grade 4 nonhematologic laboratory value if:
  Medical intervention is required to treat the subject, or
  The abnormality leads to hospitalization, or
  The abnormality persists for >1 week
5. Febrile neutropenia Grade 3 or Grade 4:
  Grade 3 is defined as ANC <1,000 mm^−3^ with a single temperature of >38.3 °C (101 °F) or a sustained temperature of ≥ 3 °C (100.4 °F) for more than 1 h
  Grade 4 is defined as ANC <1,000 mm^−3^ with a single temperature of >38.3 °C (101 °F) or a sustained temperature of ≥38 °C (100.4 °F) for more than 1 h, with life-threatening consequences and urgent intervention indicated
6. Thrombocytopenia <25,000 mm^−3^ if associated with:
  A bleeding event that does not result in hemodynamic instability but requires an elective platelet transfusion, or
  A life-threatening bleeding event which results in urgent intervention and admission to an Intensive Care Unit.
7. Prolonged delay (>2 weeks) in initiating cycle 2 due to treatment-related toxicity
8. Missing >10% of pembrolizumab doses as a result of AE(s) during the first cycle
9. Grade 5 toxicity

Treatment response was determined by serial protocolized contrast-enhanced MRI every 4 weeks for 28 weeks, and afterward at an interval of every 8 weeks for the remainder of the treatment period. Patients who completed the treatment phase entered the long-term response and survival follow-up phase of the study for the rest of life, with MRI every 16 weeks. Objective responses were evaluated by the RANO criteria^28,29^ and mRANO criteria^30^. Complete and partial responses required confirmation on the consecutive scan 4 weeks after the initial response was observed. Patients with suspected radiological progression were permitted to remain on study until progression was confirmed by follow-up MRI separated by a minimum of 4 weeks.

### Endpoints and statistical analyses

The analyses reported in this study were performed according to the statistical analysis plan. All enrolled patients were included in the safety analysis set, and patients were considered evaluable for efficacy if they received at least one dose, or part of one dose, of either study drug, had measurable tumor at baseline and completed the week 4 follow-up visit. Patients who discontinued study participation for any reason other than progressive disease or study treatment-related toxicity before the 4 week visit were not considered evaluable and were replaced; however, they continued to be monitored for safety.

The primary safety objective was to evaluate the safety of escalating doses of DNX-2401 and the overall safety of the declared dose of intratumoral DNX-2401 when followed by sequential intravenous administration of pembrolizumab. AEs and serious AEs were summarized for all patients in the study and were considered treatment related if reported as possibly, probably or definitely related to study drug.

The primary efficacy objective was to determine the objective response rate, defined as the percentage of patients that had complete or partial responses based on mRANO criteria^30^. The primary endpoint was tested in a single-arm design. As the sample size estimation was based on a prespecified historical response rate of 5%, with *α* = 0.05, a total of 39 evaluable subjects in the declared dose phase would yield an 80% power for an alternative hypothesis of objective response rate of 18%. Objective response rate was reported as the number and percentage of subjects with an objective response and the corresponding 95% CI based on the exact binomial method (Clopper–Pearson method). Type I error was set at 5% (one-sided), so it was predetermined that the 90% CI would also be provided. Secondary efficacy objectives were to evaluate 12 month overall survival as well as the clinical benefit rate, defined as the proportion of patients treated with DNX-2401 and pembrolizumab who had stable disease, complete response or partial response. Overall survival was defined as the time from the start of treatment (DNX-2401 injection) until death (or last follow-up). Overall survival at 12 months was summarized using Kaplan–Meier methods and outcomes were compared to historical rates of 20% from an approved treatment approach, NovoTTF^24^. Overall survival of patients with objective responses was compared to those without objective responses using the 6 month landmark Kaplan–Meier method to account for potential lead time bias^31^. *IDH1* mutation status and *MGMT* methylation status were assessed locally at each institution. Follow-up of survival for patients remaining alive after database lock was used for descriptive purposes only.

Statistical and computations analyses were performed using SAS 9.4 and R 4.1.3.

### Study organization and oversight

The study was conducted in compliance with the Protocol at 15 clinical trial sites in the United States and Canada, as well as recognized international standards including the Good Clinical Practice guidelines of the International Conference on Harmonisation and the principles of the Declaration of Helsinki. The Protocol and its amendments were approved by the institutional review board of each participating trial site. Voluntary written informed consent was obtained from every patient before participation in this study. DNX-2401 preparation, handling and administration followed institutional standards for biosafety level 2 agents.

### Anti-adenovirus antibodies

Anti-hexon IgG antibody levels were determined before and after treatment by ELISA from patient serum samples according to the manufacturer’s instructions (Adenovirus IgG ELISA Kit; DEIA309; Creative Diagnostics). Absorbance at 450 nm was measured using a Synergy H4 plate reader (BioTek), and concentrations calculated on the basis of a standard curve (Gen 5 software Version 3.0, BioTek). Anti-adenovirus IgG serum concentration increases of fourfold or greater were considered seroconversions. A more stringent threshold of tenfold or greater increases in levels of anti-adenovirus IgG serum concentrations was also tested.

### Targeted mutational sequencing

Targeted next-generation sequencing was performed on DNA extracted from formalin-fixed, paraffin-embedded (FFPE) pretreatment tumor biopsies available from 28 patients. Tumor samples from 18 subjects were sequenced by NeoGenomics using NeoType Discovery Profile for Solid Tumor. Tumor samples from ten subjects were sequenced by NovoGene using Novogene PM 2.0.

### Gene expression profiling and analyses

RNA was extracted from FFPE pretreatment tumor biopsies available from 38 patients and analyzed retrospectively on the NanoString nCounter system. For ten patients, there were also tumor biopsy specimens available at the time of disease progression, allowing for an examination of gene expression changes before and after treatment in matched patient samples.

The geometric mean of canonical marker genes was used to compute scores for immune cell types^32^, functional orientation markers and signature scores that are reported in this study, unless otherwise explicitly stated. Functional orientation markers and the chemokine and cytolytic signature scores were obtained from previous studies^11,33,34^. Remaining marker genes are provided in Supplementary Table 6. A T-cell-inflamed signature was computed as previously described using a weighted sum of normalized expression values of 18 inflammatory genes (CCL5, CD27, CD274 (PD-L1), CD276 (B7-H3), CD8A, CMKLR1, CXCL9, CXCR6, HLA.DQA1, HLA.DRB1, HLA.E, IDO1, LAG3, NKG7, PDCD1LG2 (PD-L2), PSMB10, STAT1 and TIGIT) related to antigen presentation, chemokine expression, cytolytic activity and adaptive immune resistance^13^. Glioblastoma microenvironment subtypes were obtained by partition-around-medoid clustering using immune cell type scores, as previously described^11^. Differentially expressed genes between groups were identified by comparing log_2_FC and Welch’s *P* values. Genes with absolute value log_2_FC >1 and *P* < 0.05 were considered differentially expressed, unless otherwise specified. Functional enrichment analysis was performed using gProfiler.

### Previously published datasets

Zhao et al. previously published their transcriptomic data in patients receiving anti-PD-1 therapy in high-grade gliomas^12^. A total of 16 patients had transcriptomic data available before initiation of anti-PD-1 therapy, and 9 patients also had transcriptomic data available at progression after initiating anti-PD-1 therapy. The transcriptomic data from these 25 patients were downloaded from SRAPRJNA482620, and clinical annotation was provided by the authors. Response was considered as stable disease or better in this study. Associations with outcome were based on overall survival after initiating anti-PD-1 therapy.

### Edema volumetric analysis

Digital Imaging and Communications in Medicine files for study MRIs were imported into Horos (version 3.3.6), and a blinded reviewer used non-motion degraded, axial, FLAIR sequences to segment perilesional FLAIR hyperintense signal. The Horos volume generator function was used to determine the total FLAIR signal volume for each study MRI. Volume of edema at each study MRI was normalized relative to baseline levels. Grouped comparisons were made by calculating the mean normalized edema volume with 95% confidence intervals at the timepoints outlined in the protocol every 4 weeks for 28 weeks and then every 8 weeks thereafter.

### IHC

We performed immunohistochemical analyses for myeloid cell markers (Iba-1, CD68 and CD163) and lymphoid cell markers (CD3, CD4 and CD8) in samples with available tissue before and after treatment in this sample. Staining and subsequent annotation and analyses were performed blinded to clinical status. Slides with 5 µm FFPE tissue sections were rehydrated and a sodium citrate-dihydrate buffer or Tris–EDTA buffer was used for heat-mediated antigen retrieval. A 3% hydrogen peroxide in methanol solution was utilized to block endogenous peroxidase activity. Blocking solution (5% bovine serum albumin in phosphate buffered saline plus 0.1% Triton X-100) was applied to slides for 1 h at room temperature. Subsequently, primary antibodies including anti-CD3 (Agilent, M725401-2, mouse monoclonal, 1:100), anti-IBA1 (Wako, 019-19741, rabbit polyclonal, 1:1,500), anti-CD68 (Agilent, M0514, mouse monoclonal, 1:200), anti-CD4 (abcam, ab133616, rabbit monoclonal, 1:100) and anti-CD8 (abcam, ab93278, rabbit monoclonal, 1:250) were applied overnight at 4 °C in blocking solution. A 1 h incubation with secondary antibody was performed followed by processing with the DAKO polymer-HRP system and DAB peroxidase kit, counterstaining with hematoxylin, dehydration of the tissue and coverslipping. Whole slide images were digitized, and then for each slide tumor versus non tumor content was annotated and representative images were selected. Proportions of stain-positive cells were quantified using HALO (version 3.0311, Indica Labs) software algorithms that were defined to identify cells with either nuclear or cytoplasmic staining as a fraction of all cells. This algorithm was applied to all annotated tissue sections in an unbiased systematic manner, and the density of immunopositivity per square millimeter was recorded for each antibody. PD-L1 protein expression was performed by NeoGenomics Laboratories (NeoGenomics) under the direction of Merck using FFPE tumor biopsy samples according to standard protocols (PD-L1 IHC 22C3 assay).

### Multiplex immunofluorescence staining, tissue imaging and cell phenotyping

A validated and standardized multiplex immunofluorescence protocol was developed for simultaneous detection of CD3, CD8, CD11b, CD163, GFAP and DAPI in a single FFPE tissue section. The validation pipeline for the multiplex immunofluorescence protocol has been previously described by our group^8^. Briefly, whole-slide tissue sections were deparaffinized and subjected to sequential rounds of antibody staining. Antigen retrieval was performed using Dako PT-Link heat-induced antigen retrieval with low pH (pH 6) or high pH (pH 9) target retrieval solution (Dako). The antibody panel included CD11b (rabbit monoclonal, clone EPR1344, 1:1,000, Abcam, product number ab133357), CD163 (mouse monoclonal, clone MRQ-26, ready-to-use, Cell Marque, product number 760-4437), CD3 (rabbit polyclonal, IgG, ready-to-use, Agilent, product number IR503), CD8 (mouse monoclonal, clone C8/144B, ready-to-use, Agilent, product number IR623), and GFAP (mouse monoclonal, clone 6F2, 1:500, Agilent, product number M0761). After all sequential rounds, nuclei were counterstained with spectral DAPI (Akoya Biosciences) and sections were mounted with Faramount Aqueous Mounting Medium (Dako).

Multiplexed immunofluorescence slides were scanned on a Vectra-Polaris Automated Quantitative Pathology Imaging System (Akoya Biosciences). Spectral unmixing was performed using inForm software (version 2.4.8, Akoya Biosciences), as described. Image analysis was performed using QuPath and Fiji/ImageJ. Briefly, cells were segmented on the basis of nuclear detection using the StarDist 2D algorithm. A random trees algorithm classifier was trained for each cell marker. Cells were then subclassified as CD3^+^, CD8^+^, CD11b^+^ and CD163^+^ cells. CD4^+^ T cells were defined as CD3^+^ CD8^−^. Cells negative for these markers were defined as ‘other cell types’. Measurements were calculated as cell densities (cells mm^−2^). GFAP was used to identify tumor areas.

### Reporting summary

Further information on research design is available in the Nature Portfolio Reporting Summary linked to this article.

## Online content

Any methods, additional references, Nature Portfolio reporting summaries, source data, extended data, supplementary information, acknowledgements, peer review information; details of author contributions and competing interests; and statements of data and code availability are available at 10.1038/s41591-023-02347-y.

## Supplementary information

Supplementary Information Supplementary Methods, Tables 1–6 and Figs. 1 and 2.

Reporting Summary

## Figures and Tables

**Extended Data Table 1:**
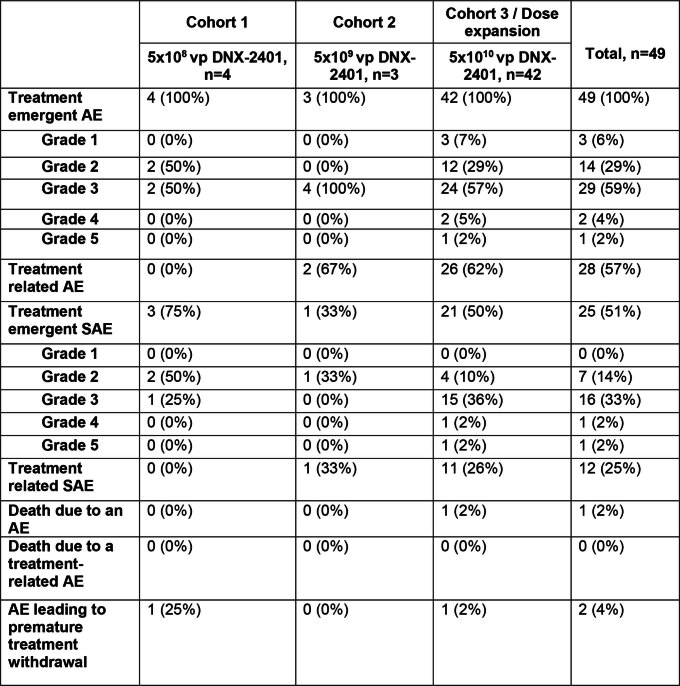
Summary of adverse events

AE denotes adverse event, SAE denotes serious adverse event; frequency refers to patients reporting at least one adverse event (each patient is included once), treatment related include those classified as possibly, probably, or definitely related to DNX2401 + pembrolizumab.

**Extended Data Table 2:**
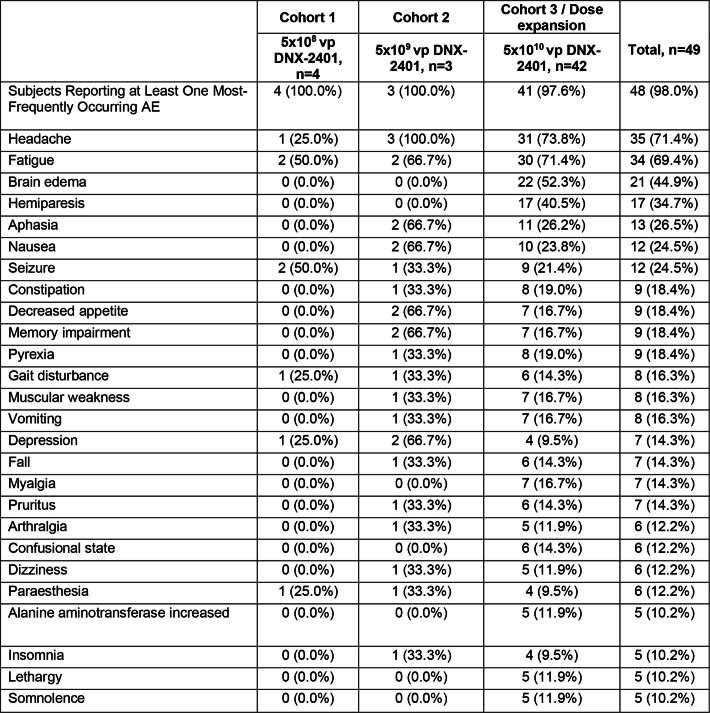
Summary of most commonly reported adverse events

AE denotes adverse event. Listed are the most frequently reported AEs (≥ 10% Overall) by decreasing frequency of preferred term.

**Extended Data Table 3:**
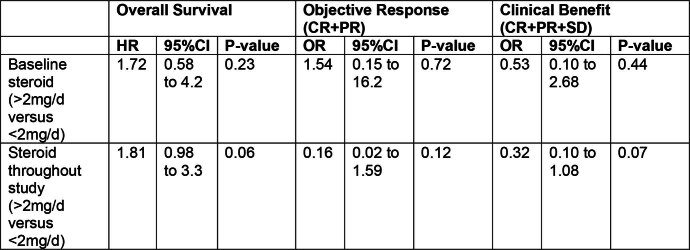
Corticosteroid use and outcomes

HR denotes Hazard Ratio, OR denotes Odds Ratio, CI denotes confidence interval, CR denotes complete response; PR denotes partial response, SD denotes stable disease.

## Data Availability

Pseudononymized participant data, including outcomes and relevant reported patient characteristics, are shared as Supplementary Information. Processed gene expression data that can be linked to pseudonymized participant data are provided at GSE226976. Previously published data were accessed from SRAPRJNA482620 with clinical annotation provided from authors. Custom algorithms or software were not used to generate the results reported in this manuscript.
