# Author Correction: Oncolytic DNX-2401 virotherapy plus pembrolizumab in recurrent glioblastoma: a phase 1/2 trial

**DOI:** 10.1038/s41591-025-03895-1

**Published:** 2025-07-21

**Authors:** Farshad Nassiri, Vikas Patil, Leeor S. Yefet, Olivia Singh, Jeff Liu, Rachel M. A. Dang, Takafumi N. Yamaguchi, Mariza Daras, Timothy F. Cloughesy, Howard Colman, Priya U. Kumthekar, Clark C. Chen, Robert Aiken, Morris D. Groves, Shirley S. Ong, Rohan Ramakrishna, Michael A. Vogelbaum, Simon Khagi, Thomas Kaley, Jason M. Melear, David M. Peereboom, Analiz Rodriguez, Maxim Yankelevich, Suresh G. Nair, Vinay K. Puduvalli, Kenneth Aldape, Andrew Gao, Álvaro López-Janeiro, Carlos E. de Andrea, Marta M. Alonso, Paul Boutros, Joan Robbins, Warren P. Mason, Adam M. Sonabend, Roger Stupp, Juan Fueyo, Candelaria Gomez-Manzano, Frederick F. Lang, Gelareh Zadeh

**Affiliations:** 1 Division of Neurosurgery, University of Toronto, Toronto, Ontario Canada; 2 Princess Margaret Cancer Center, University Health Network, Toronto, Ontario Canada; 3 Department of Human Genetics, University of California Los Angeles, Los Angeles, CA USA; 4 Division of Neuro-oncology, University of California San Francisco, San Francisco, CA USA; 5 UCLA Neuro-Oncology Program, David Geffen School of Medicine, University of California Los Angeles, Los Angeles, CA USA; 6 Huntsman Cancer Institute and Department of Neurosurgery, University of Utah, Salt Lake City, UT USA; 7 Department of Neurology, Division of Neuro-Oncology, Northwestern University Feinberg School of Medicine, Chicago, IL USA; 8 Department of Neurosurgery, University of Minnesota, Minneapolis, MI USA; 9 Rutgers Cancer Institute of New Jersey, Rutgers University, New Brunswick, NJ USA; 10 Department of Neurology, Texas Oncology, Austin, TX USA; 11 Division of Neuro-Oncology, Department of Neurology, the Ohio State University Wexner Medical Center, Columbus, Ohio USA; 12 Department of Neurological Surgery, Weill Cornell Medical College, New York Presbyterian Hospital, New York, NY USA; 13 Department of Neuro-Oncology, Neuro-Oncology Program, Moffitt Cancer Center, Tampa, FL USA; 14 Division of Medical Oncology, University of North Carolina at Chapel Hill, Chapel Hill, NC USA; 15 Department of Neurology, Memorial Sloan Kettering Cancer Center, New York, NY USA; 16 Department of Internal Medicine, Baylor University Medical Center, Dallas, TX USA; 17 The Rose Ella Burkhardt Brain Tumor and Neuro-Oncology Center, Cleveland Clinic, Cleveland, OH USA; 18 Department of Neurosurgery, University of Arkansas for Medical Sciences, Little Rock, AK USA; 19 Department of Pediatrics, University of Michigan, Ann Arbor Beaumont Children’s Hospital, Royal Oak, MI USA; 20 Lehigh Valley Topper Cancer Institute, Allentown, PA USA; 21 Department of Neuro-Oncology, The University of Texas MD Anderson Cancer Center, Houston, TX USA; 22 Laboratory of Pathology, National Cancer Institute, Bethesda, MD USA; 23 Department of Laboratory Medicine and Pathobiology, University Health Network, Toronto, Ontario Canada; 24 Department of Pathology, Clínica Universidad de Navarra, Pamplona, Spain; 25 Navarra Institute for Health Research (IdISNA), Pamplona, Spain; 26 Department of Pediatrics, Clínica Universidad de Navarra, Pamplona, Spain; 27 Program of Solid Tumors, Center for the Applied Medical Research (CIMA), Pamplona, Spain; 28 DNATrix Inc., Carlsbad, CA USA; 29 Department of Neurological Surgery, Feinberg School of Medicine, Northwestern University, Chicago, IL USA; 30 Northwestern Medicine Malnati Brain Tumor Institute of the Lurie Comprehensive Cancer Center, Feinberg School of Medicine, Northwestern University, Chicago, IL USA; 31 Department of Medicine, Division of Hematology/Oncology, Feinberg School of Medicine, Northwestern University, Chicago, IL USA; 32 Department of Neurology, Feinberg School of Medicine, Northwestern University, Chicago, IL USA; 33 Department of Neurosurgery, The University of Texas MD Anderson Cancer Center, Houston, TX USA; 34 Department of Surgery, University of Toronto, Toronto, Ontario Canada

**Keywords:** CNS cancer, Phase II trials

Correction to: *Nature Medicine* https://doi.org/10.1038/s41591-023-02347-y, published online 15 May 2023.

In the version of the article initially published, in the “TME-high” row in Extended Data Fig. 6a, the images for “CD3” and “CD8” were identical. Extended Data Fig. 6a has now been amended with the correct image for “CD8” in the HTML and PDF versions of the article.

