# Supplementary material for: Oncolytic DNX-2401 virotherapy plus pembrolizumab in recurrent glioblastoma: a phase 1/2 trial
Source: Nat Med. 2023 May 15;29(6):1370–8. doi: 10.1038/s41591-023-02347-y (PMC10287560; doi:10.1038/s41591-023-02347-y)
Supplement: Supplementary file 1 — Supplementary Methods, Tables 1–6 and Figs. 1 and 2. [file 41591_2023_2347_MOESM1_ESM.pdf]

nature medicine

Article https://doi.org/10.1038/s41591-023-02347-y

Oncolytic DNX-2401 virotherapy plus pembrolizumab in recurrent glioblastoma: a phase 1/2 trial

In the format provided by the authors and unedited

Supplementary Methods:

Inclusion Criteria:

1. ≥ 18 years of age on the day of informed consent
2. A single glioblastoma or gliosarcoma tumor confirmed by documented historical histopathology.
    1. First or presenting second recurrence of glioblastoma or gliosarcoma (i.e., relapse following prior treatment) at time of consent. Approval may be given by Medical Monitor or designee to proceed with enrollment with a prior non-GBM/GS diagnosis, in which case transition to GBM/GS may be accepted as first recurrence of tumor.
    2. Gross total or partial tumor resection, including tumor debulking, is not possible or not planned
    3. A single measurable tumor that is at least 10.0 mm longest diameter (LDi) x 10.0 mm shortest diameter (SDi) and that does not exceed 40.0 mm in LDi or SDi on the Screening MRI
    4. The measurable area of the tumor is solid/nodular and is not cystic
    5. Willing to provide a stereotactic biopsy sample from the brain tumor obtained prior to DNX- 2401 administration
    6. Tumor must be accessible for stereotactic injection
    7. Evidence of tumor recurrence (e.g., progression after last treatment) on the Screening MRI (15 days to 72 hours prior to DNX-2401 administration)
    8. Tumor location that will not risk delivery of DNX-2401 into the ventricular system

3. Tumor recurrence or progression after previously failing surgical resection, chemotherapy or radiation
4. Resolution of toxic effect(s) of the most recent prior chemotherapy to Grade 1 or less (except neuropathy and alopecia)
5. Demonstrate adequate organ function as defined below:
    - Hematological
        1. Absolute neutrophil count (ANC) ≥ 1,500 cells/mm^3
        2. WBC ≥ 2.5 x 10^3 cells/mm^3
        3. Platelets ≥ 100,000 cells/mm^3
        4. Hemoglobin ≥ 10 g/dL or ≥ 5.6 mmol/L
        5. Absolute lymphocyte count (ALC) ≥ 800 cells/mm^3
    - Renal
        1. Creatinine ≤ 1.5x ULN
        2. BUN ≤1.5x ULN
    - Hepatic
        1. Total bilirubin ≤ 1.5x upper limit of normal (ULN)
            1. **Note:** In the event that total bilirubin is > 1.5 X ULN, the subject may be eligible if the direct bilirubin level is ≤ ULN, following consultation with the DNAtrix Medical Monitor or designee.
        2. AST (SGOT) and ALT (SGPT) ≤ 2.5x ULN
    - Coagulation
        1. International Normalized Ratio (INR) ≤1.5x ULN
        2. Prothrombin Time (PT) ≤ 1.5x ULN

    3. Activated Partial Thromboplastin Time (aPTT) ≤ 1.5x ULN
6. Adequate venous access
7. Karnofsky performance status ≥ 70%
8. Afebrile at baseline/Day 0 prior to DNX-2401 administration (i.e., < 38.0°C)
9. Prior anti-tumor therapies must have been completed within the following time periods prior to DNX-2401 injection:

- 2 weeks after vincristine
- 4 weeks after nitrosoureas
- 3 weeks after procarbazine or temozolomide
- 4 weeks after bevacizumab, other antibody therapy or other anti-angiogenic therapy to treat glioblastoma
- 5 half-lives for other anti-cancer agents or 2 weeks after the last dose when the half-life is unknown. A discussion of these agents will take place with the DNAtrix Medical Monitor or designee prior to establishing eligibility.

10. For applicable screening candidates, external beam radiotherapy (> 5000 cGy) must have been completed at least 12 weeks prior to DNX-2401 administration
11. Females who are not of childbearing potential must be documented as such and will not be tested for pregnancy or required to utilize contraception if they meet one or more of the following definitions of non-childbearing potential:
    - Amenorrheic for > 2 years without a hysterectomy and bilateral oophorectomy and a FSH value in the postmenopausal range upon pre-trial (screening) evaluation
    - Post-hysterectomy, bilateral oophorectomy or tubal ligation. Tubal ligation must be confirmed with medical records of the actual procedure.
12. Female subjects of childbearing potential must have a negative urine or serum pregnancy test within 24 hours prior to receiving DNX-2401 injection. If the urine test is positive or cannot be confirmed as negative, a serum pregnancy test will be required to confirm negative results. The serum pregnancy test must be negative for the subject to be eligible.
13. Female subjects of childbearing potential must be willing to use two highly effective birth control methods throughout the study, starting with provision of informed consent through 180 days after the single dose of DNX-2401 and 120 days after the last dose of pembrolizumab. The two birth control methods can be either two barrier methods or a barrier method plus a hormonal method to prevent pregnancy.

    Examples of highly effective birth control methods include the following:

- Using twice the normal protection of birth control (i.e., double-barrier) by using a condom AND spermicidal jelly or foam, or a diaphragm AND spermicidal jelly or foam. A spermicidal jelly or foam must be used in addition to a barrier method (e.g., condom or diaphragm)
- Oral contraceptive control pills
- Depot or injectable birth control
- Intrauterine Device (IUD)
- Transdermal contraceptive patch
- Vaginal contraceptive ring

14. Male subjects must agree to use an acceptable method of contraception throughout the study starting with provision of informed consent through 180 days after the single dose of DNX- 2401 and 120 days after the last dose of pembrolizumab.
15. Willing and able to provide informed consent, undergo and comply with all study assessments and adhere to the protocol schedule
16. Agree not to donate blood or gametes following DNX-2401 administration

Exclusion Criteria:

Subjects who meet any of the following exclusion criteria are not eligible for the study and must not be enrolled:

1. Recurrent GBM with multiple (> 2) separate enhancing tumors (measurable or non-measurable)
2. Tumor shape that is bi-lobular or multifocal tumor
3. Tumor involvement that would require ventricular, brainstem or posterior fossa injection or access through a ventricle or risk of ventricular penetration in order to deliver DNX-2401
4. Tumor involves both hemispheres or there is suspected cerebrospinal fluid (CSF) dissemination
5. Documented extracranial metastases
6. Requires, or based upon historical evidence, may require treatment with high-dose systemic corticosteroids defined as dexamethasone > 4 mg/day or bioequivalent for more than 3 consecutive days within 2 weeks prior to and following the first dose of pembrolizumab, or has demonstrated an inability to be tapered off of steroids
7. Uncontrolled blood-sugar levels defined as HbA1c > 7%
8. Active autoimmune disease that requires, or has required, systemic treatment in the past 2 years (i.e. with use of disease modifying agents, corticosteroids or immunosuppressive drugs)

9. Previous treatment with any checkpoint inhibitor (e.g., anti-PD-1, anti-PD-L1, or anti-PD-L2 agent) or with an agent directed to another stimulatory or co-inhibitory T-cell receptor (e.g., CTLA-4, OX-40, CD137), including pembrolizumab
10. History of (non-infectious) pneumonitis that required steroids or current pneumonitis
11. History of interstitial lung disease
12. Transfusions or medications (e.g., G-CSF) to treat pancytopenia or other hematological conditions within 4 weeks prior to DNX-2401 administration
13. Prior gene transfer therapy or prior therapy with cytolytic virus of any type
14. Live vaccines of any kind within 45 days prior to DNX-2401 administration and while participating in the study. Examples of live vaccines include, but are not limited to, the following: measles, mumps, rubella, varicella/zoster (chicken pox), yellow fever, rabies, BCG, and typhoid vaccine. Seasonal influenza vaccines for injection are generally killed virus vaccines and are permitted; however, intranasal influenza vaccines (e.g. Flu-Mist®) are live attenuated vaccines and are not allowed.
15. Major surgery within 4 weeks and minor surgery within 2 weeks of DNX-2401 administration (Refer to Appendix 2 – Major and Minor Surgery Definitions)
16. Participation in an investigational (invasive device, drug, or product) study or treatment with an investigational agent or device within 30 days prior to consent

17. Any contraindication for undergoing MRI such as: individuals with pacemakers, epicardial pacer wires, infusion pumps, surgical and/or aneurysm clips, shrapnel, metal prosthesis, implants with potential magnetic properties, or metallic bodies in the eyes
18. Is pregnant or breastfeeding, or planning to conceive or father children during the study, starting with the screening visit through 180 days after the single dose of DNX-2401 and 120 days after the last dose of pembrolizumab
19. Evidence of active uncontrolled infection or an unstable or severe intercurrent medical condition that requires treatment and/or precludes surgery
20. History of prior malignancy except for curatively treated basal or squamous cell carcinoma of the skin (non-melanoma skin cancer), cervical or vaginal intra-epithelial neoplasia, non- invasive breast cancer *in situ* or localized prostate cancer with a prostate specific antigen (PSA) of < 4.0 ng/mL (mcg/L) at Screening. Subjects with other curatively treated malignancies who had no evidence of metastatic disease and a > 2 year disease-free interval may be enrolled after approval by the DNAtrix Medical Monitor or designee.
21. Any medical condition that precludes intratumoral injection into the brain

22. Immunocompromised subjects or those with autoimmune conditions, human immunodeficiency virus (HIV), or active hepatitis (according to diagnostic serology results that are positive for active HAV, HBV [Anti-HBc and HBsAg] or HCV infection)
23. Pulmonary conditions including a known history of active tuberculosis (TB, *Mycobacterium tuberculosis*). TB testing is required for subjects recently exposed to persons with active TB or who have traveled recently to areas where TB is endemic.
24. Evidence of bleeding diathesis, hemorrhage, or coagulopathy or use of anticoagulant medication or any medication that may increase the risk of bleeding that cannot be stopped prior to surgery. If the medication can be discontinued prior to DNX-2401 injection, then the subject may be eligible following consultation with the DNAtrix Medical Monitor or designee.
25. Encephalitis, multiple sclerosis or other central nervous system (CNS) infection or primary CNS disease that would interfere with subject evaluation
26. Li-Fraumeni Syndrome or with a known germ line deficit in the retinoblastoma gene or its related pathways
27. Significant systemic or major illnesses including, but not limited to, congestive heart failure, ischemic heart disease, kidney disease or renal failure, organ transplantation or other conditions that may affect subject risk or protocol compliance
28. Alcohol or substance abuse or alcohol dependency within 12 months prior to screening that has caused health consequences
29. History or current diagnosis of any medical or psychological condition, and in particular, any unstable CNS condition such as delirium, confusion, etc., that might interfere with the subject’s ability to comply with the study requirements or the ability to obtain informed consent

Supplementary Table 1. Summary of adverse events by counts

	Cohort 1	Cohort 2	Cohort 3 / Dose expansion	Total, n=49
	$5x10^{8}$ vp DNX-2401, n=4	$5x10^{9}$ vp DNX-2401, n=3	$5x10^{10}$ vp DNX-2401, n=42	
**Total number of treatment emergent AE**	26	78	672	776
**Grade 1**	8	41	389	438
**Grade 2**	13	26	205	244
**Grade 3**	5	11	73	89
**Grade 4**	0	0	4	4
**Grade 5**	0	0	1	1
**Total number of treatment emergent SAE**	5	1	43	49
**Grade 1**	0	0	1	1
**Grade 2**	3	1	11	15
**Grade 3**	2	0	27	29
**Grade 4**	0	0	3	3
**Grade 5**	0	0	1	1

Shown are total counts of adverse events, AE denotes adverse event, SAE denotes serious adverse event

Supplementary Table 2. Bevacizumab use during treatment phase.

**Subject ID**	**Start Date (study day)**	**End date (study day)**	**Dose**	**Unit**	**Frequency**	**Indication**
2401007	54	54	735	ml	once	Prophylaxis: edema
2401023	32	60	5	mg/kg	every 2 weeks	Adverse event: vasogenic edema
2401025	37	63	5	mg/kg	every 2 weeks	Adverse event: vasogenic edema
	98	126	5	mg/kg	every 2 weeks	Adverse event: vasogenic edema
2401026	43	70	3	mg/kg	every 2 weeks	Adverse event: vasogenic edema
	103	147	5	mg/kg	every 2 weeks	Adverse event: vasogenic edema
2401036	103	103	410	mg	once	Adverse event: left sided weakness
	122	122	420	mg	once	Adverse event: left sided weakness
	145	145	420	mg	once	Adverse event: left sided weakness
2401037	82	82	340	mg	once	Adverse event: vasogenic edema
	93	93	340	mg	once	Adverse event: vasogenic edema
	109	109	340	mg	once	Adverse event: vasogenic edema
2401038	60	60	330	mg	once	Adverse event: vasogenic edema
	81	81	330	mg	once	Adverse event: vasogenic edema
	102	102	330	mg	once	Adverse event: vasogenic edema
2401040	54	54	5	mg/kg	once	Adverse event: vasogenic edema
	71	71	5	mg/kg	once	Adverse event: vasogenic edema
	85	85	5	mg/kg	once	Adverse event: vasogenic edema
	97	97	10	mg/kg	once	Adverse event: right sided weakness
2401041	75	75	5	mg/kg	once	Adverse event: vasogenic edema
	96	96	5	mg/kg	once	Adverse event: vasogenic edema
	173	173	10	mg/kg	once	Adverse event: vasogenic edema
2401042	135	135	720	mg	once	Adverse event: vasogenic edema
	149	149	370	mg	once	Adverse event: vasogenic edema
	164	164	370	mg	once	Adverse event: vasogenic edema
2401043	175	175	320	mg	once	Other: Progression of disease
	189	189	320	mg	once	Other: Progression of disease
	203	203	320	mg	once	Other: Progression of disease
	280	280	640	mg	once	Other: Progression of disease
	293	293	640	mg	once	Other: Progression of disease
	306	306	320	mg	once	Other: Progression of disease
2401048	52	52	385	mg	once	Adverse event: vasogenic edema
	66	66	385	mg	once	Adverse event: vasogenic edema
	80	80	385	mg	once	Adverse event: vasogenic edema

**Supplementary Table 3**. Details of serious adverse events attributable to study treatment							
**Adverse Event** / **Subject ID**	**Dose DNX-2401 (vp)**	**Days after DNX-2401 injection**	**CTCAE Grade**	**Treatment**	**Effect on DNX-2401 or Pembrolizumab**	**Outcome**	**Sequalae**
Dysphasia							
2401008	$5x10^9$	29-41	2	Steroids	None	Resolved	Yes
2401025	$5x10^{10}$	1-4	2	None	None	Resolved	None
2401029	$5x10^{10}$	9-10	2	Steroids	None	Resolved	None
Headache							
2401009	$5x10^{10}$	17-24	2	Concomitant medication	None	Resolved	None
2401031	$5x10^{10}$	10-12	2	Concomitant medication	None	Resolved	None
Edema							
2401015	$5x10^{10}$	24-28	3	Steroids	Interrupted	Resolved	None
2401018	$5x10^{10}$	11-20	3	Steroids	None	Resolved	None
2401037	$5x10^{10}$	65-66	3	Concomitant medication and steroids	None	Resolved	Yes
2401040	$5x10^{10}$	53-55	3	Concomitant medication and steroids	Interrupted	Resolved	Yes
2401042	$5x10^{10}$	132-135	4	Concomitant medication	Interrupted	Resolved	None
2401044	$5x10^{10}$	39-44	3	Steroids	None	Resolved	None
2401044	$5x10^{10}$	92-96	3	Concomitant medication	Interrupted	Resolved	Yes
2401047	$5x10^{10}$	10-12	2	Concomitant medication	None	Resolved	None
2401048	$5x10^{10}$	23-24	3	Steroids	Discontinued	Resolved	Yes
Hemiparesis							
2401025	$5x10^{10}$	1-4	3	None	None	Resolved	None
2401029	$5x10^{10}$	9-10	3	Steroids	None	Resolved	Yes
2401048	$5x10^{10}$	23-26	3	Steroids	Discontinued	Resolved	Yes
Autoimmune Colitis							
2401022	$5x10^{10}$	87-92	3	Concomitant medication	Discontinued	Resolved	None
Noninfectious Meningitis							
2401045	$5x10^{10}$	4-9	3	Concomitant medication	None	Resolved	None
Somnolence							
2401048	$5x10^{10}$	23-24	2	Concomitant medication	Discontinued	Resolved	None

Supplementary Table 4. Subsequent therapies after trial termination

**Therapies**	**Declared dose population Patients, No. (%)**	**Intent-to-treat population Patients, No. (%)**
Any subsequent therapy	30 (71)	36 (75)
Surgery	10 (24)	13 (27)
Laser ablation	1 (2)	1 (2)
Radiotherapy	9 (21)	11 (23)
Systemic Therapy		
Bevacizumab	21 (50)	23 (48)
Lomustine	13 (31)	17 (35)
Pembrolizumab	7 (17)	8 (16)
Temozolomide	6 (14)	7 (15)
Carboplatin	2 (5)	3 (6)
Everolimus	2 (5)	2 (5)
Imipridone	2 (5)	2 (5)
Depatuxizumab mafodotin	2 (5)	2 (5)
Procarbazine	1 (2)	1 (2)
Etoposide	1 (2)	1 (2)
Olaparib	1 (2)	1 (2)
Pabinostat	1 (2)	1 (2)
Irinotecan	1 (2)	1 (2)
Terameprocol	1 (2)	1 (2)
Debio1347	0 (0)	1 (2)
Nivo	0 (0)	1 (2)
Dabrafenib	0 (0)	1 (2)
Trametinib	0 (0)	1 (2)

Supplementary Table 5. Molecular features with potential clinical relevance identified in pretreatment tumor biopsies

DNX-2401 dose (vp)	Subject ID	Best Response	Investigator Reported Molecular Features[2]					Targeted Sequencing Identified Molecular Features[3]
			PD-L1[1]	MGMT	IDH1	IDH2	Other	Selected mutations
**5e8**	2401001	SD	POS	UNM	WT	WT	MUT=1P<br>WT=19Q	MUT=TP53 92%<br>WT=IDH1, PTEN, RB1, NF1, MTOR, POLE
	2401003	SD	POS	UNM	WT	WT	MUT= GFAP, Vimentin, EGFR, OTIG2, PhosphoH3<br>KLE7 20%, TP53 rare positive cells	MUT=PTEN 55%<br>WT=IDH1, RB1, TP53, NF1, MTOR, POLE
	2401049	PD	NR	MET	WT	NR	MUT=EGFR, GFAP<br>WT=KI67, P53, PTEN, VEGF	WT=IDH1, PTEN, RB1, TP53, NF1, MTOR, POLE
	2401011	PD	NEG	UNM	WT	WT	MUT=EGFR, GFAP<br>WT=EGFRVIII, KI67, P53<br>ATRX retained	MUT=RB1 38%<br>WT=IDH1, PTEN, NF1, TP53, MTOR, POLE
**5e9**	2401002	PD	POS	NR	MUT	NR	NR	MUT=IDH1 43%<br>WT=PTEN, RB1, TP53, NF1, MTOR, POLE
	2401008	SD	NEG	UNM	WT	NR	MUT=P53, EGFR	MUT=TP53 69%<br>WT=IDH1, PTEN, RB1, TP53, NF1, MTOR, POLE
	2401012	SD	NR	MET	WT	NR	MUT=BRAF	MUT=POLE 24%<br>WT=IDH1, PTEN, RB1, TP53, NF1, MTOR
**5e10**	2401004	PD	POS	NR	MUT	WT	MUT=ATRX, PDGFRA, RAD50, RB1 loss, TP53	MUT=IDH1 44%, PT53 93%<br>WT=RB1, TP53, NF1, MTOR, POLE

2401005	SD	NEG	UNM	WT	WT	MUT=CDK4, ERBB3, FRS2, MDM2, SETD2	NR
2401006	SD	POS	MET	WT	NR	MUT=EGFR, SETD2, TERT<br>MSS, TMB-LOW	WT=IDH1, PTEN, RB1, TP53, NF1, MTOR, POLE
2401007	PD	NEG	UNM	WT	WT	MUT=EGFR, P53	NR
2401009	PD	NEG	UNM	WT	WT	MUT=EGFR (diffuse)<br>WT=P53	NR
2401010	PD	NR	UNM	WT	WT	MUT=EGFR, P53<br>KI67 26%	NR
2401013	CR	NR	NR	WT	NR	NR	MUT= NF1 16%, IDH2 23%<br>WT=IDH1, PTEN, RB1, TP53, MTOR, POLE
2401014	SD	POS	NR	WT	NR	MUT=P53<br>ATRX retained	MUT=PTEN 72%, TP53 71%, MTOR 40%<br>WT=IDH1, RB1, NF1, POLE
2401015	PD	NR	MET	WT	NR	NR	WT=IDH1, PTEN, RB1, TP53, NF1, MTOR, POLE
2401016	PD	POS	NR	WT	NR	NR	NR
2401017	SD	POS	NR	WT	NR	NR	MUT=PTEN 21%<br>WT=IDH1, RB1, TP53, NF1, MTOR, POLE
2401018	PD	POS	UNM	WT	NR	NR	MUT=NF1 29%, MTOR 49%, POLE 77%<br>WT=IDH1, PTEN, RB1, TP53
2401019	PR	POS	MET	WT	NR	NR	MUT=IDH1 12%, TP53 29%<br>WT=RB1, NF1, MTOR, POLE
2401020	SD	POS	UNM	WT	NR	WT=P53	NR
2401021	SD	NEG	MET	WT	NR	MUT=EGFR, EGFRVIII<br>KI67 >50%	NR
2401022	PD	NR	UNM	MUT	NR	MUT=P53	NR

						KI67 >25%	
2401023	SD	NEG	MET	WT	WT	MUT=GFAP, P53 (IHC), RB1, BRCA1, BRCA2, MYCN, PTEN 89%, TP53 89%<br>WT=1P/19Q, ATRX, BRAF, EGFR,	MUT=PTEN 27%, RB1 25%, TP53 25%, NF1 23%, MTOR 18%, POLE 17%
2401024	PD	NEG	MET	WT	WT	MUT=CDK4, EGFR, MDM2<br>KI67 HIGH	MUT=PTEN 58%<br>WT=IDH1, RB1, TP53, NF1, MTOR, POLE
2401025	SD	NR	UNM	WT	WT	MUT=GFAP, ATM, CDKN2A, PTEN, RB1, TERT, TP53<br>KI67 25-30%<br>WT=EGFRVIII	MUT=PTEN 34%, TP53 33%<br>WT=IDH1, RB1, NF1, MTOR, POLE
2401026	SD	NEG	MET	WT	WT	MUT=PTEN<br>KI67 10-15%<br>WT=1P/19Q, EGFR, P53	NR
2401027	SD	NEG	UNM	WT	WT	MUT=EGFR<br>KI67 20%<br>WT=P53	NR
2401028	PD	POS	MET	NR	NR	MUT=GFAP, P53<br>KI67 60%	NR
2401029	SD	POS	UNM	WT	WT	MUT=22Q, ATRX, DDX3X, NF1, PTEN, TP53	MUT=PTEN 40%, RB1 45%, TP53 41%, NF1 41%<br>WT=IDH1, MTOR, POLE
2401030	PD	POS	UNM	WT	WT	MUT=EGFRVIII, PTEN, TERT	NR
2401031	SD	NR	UNM	WT	WT	MUT=CDKN2A, PTEN, EGFRVIII	WT=IDH1, PTEN, RB1, TP53, NF1, MTOR, POLE
2401032	SD	NEG	UNM	WT	WT	MUT=CDKN2A, CDKN2B, CREBBP, MYCN, PTEN, TERT, TP53	MUT=TP53 15%<br>WT=IDH1, PTEN, RB1, NF1, MTOR, POLE

2401033	SD	NEG	UNM	WT	NR	MUT=CDKN2A/B, EGFRVIII, ERRFI1, TERT	WT=IDH1, PTEN, RB1, TP53, NF1, MTOR, POLE
2401034	PD	NR	UNM	WT	WT	MUT=EBFRVIII, GFAP, P53 5%<br>KI67 30%<br>WT=1P/19Q	NR
2401035	PD	NR	UNM	WT	WT	MUT=CDK4, CDKN2AP16INK4A, CDKN2B, MDM2, PTEN	NR
2401036	PD	NEG	UNM	WT	WT	MUT=ARID5B, CDKN2A, CDKN2B, EGFR, NF1, SUFU, TERT 41%	NR
2401037	PD	POS	UNM	WT	WT	MUT=1P36/1Q24, ANKRD11 6%, CDKN2AP14ARF, CDKN2AP16INK4A, CDKN2B, EGFR, FOXA1 39%, GATA3, IKBKE, IL10, KMT2B 26%, MDM4, TERT 36%	NR
2401038	PD	POS	MET	WT	WT	MUT=CCND2, CDK4, EGFR, EIF1AX, FAM58A, GLI1, MDM2, SDHD, TERT	NR
2401039	PR	NEG	UNM	WT	WT	MUT= CDKN2A/B, EGFRVIII, MDM4, NF1, PIK3CB, RUNX1, TERT	WT=IDH1, PTEN, RB1, TP53, NF1, MTOR, POLE
2401040	PD	NEG	UNM	MUT	WT	MUT=ATRX 59%, BAP1, BLM 19%,<br>CDKN2AP14ARF, CDKN2AP16INK4A, CDKN2B, ERCC3 6%, MAP2K4 16%, MED12 9%, MLH1, PBRM1, PDGFRA, RAD51C 9%, SETD2, SPEN 25%, TERT 25%, TGFBR2, TP53 exon5 46%, exon 7 47%	MUT=IDH1 30%, TP53 44%, NF1 21%, MTOR 33%<br>WT=PTEN, RB1, POLE
2401041	SD	NR	UNM	WT	NR	MUT= EGFR, CDK4, MDM2, PDGFRA, PTEN 25%, TERT 22%, 19%	NR

2401042	PD	NEG	MET	WT	WT	MUT= CCND2, CDK4, EGFR, MET, SOX2, TERT, TP53	NR
2401043	PD	NR	MET	WT	WT	WT=BRAF, EGFR, PTEN	NR
2401044	SD	NR	UNM	WT	WT	MUT= glioma mutation panel<br>KI67 14%<br>WT=H3K27M	NR
2401045	CR	NR	MET	WT	WT	WT=BRAF, TP53	MUT=TP53 16%<br>WT=IDH1, PTEN, RB1, NF1, MTOR, POLE
2401046	SD	NEG	UNM	WT	WT	MUT=TP53, ATRX, H3F3AK27M, NF1, PIK3CA, RB1	MUT=TP53 77%, NF1 44%, POLE 57%<br>WT=IDH1, PTEN, RB1, MTOR
2401047	PR	NEG	NR	WT	NR	MUT=H3K27M<br>ATRX retained	WT=IDH1, PTEN, RB1, TP53, NF1, MTOR, POLE
2401048	SD	NR	UNM	WT	WT	MUT=GFAP, H3K27ME, TP53, Y236H<br>WT=H3K27M	NR

Assay and analysis performed by Neogenomics Inc. as directed by Merck; 2. Various assays used; 3. Targeted NGS using NeoType Discovery Profile for Solid Tumors (NeoGenomics) and/or Novogene PM2.0.(Novogene); Abbreviations: ; CR=complete response, PD=progressive disease, PR=partial response, SD=stable disease; MET=MGMT promoter methylation present/positive (methylated, hypermethylated), UNM=MGMT promoter methylation absent/negative (unmethylated), MUT= mutated/amplified/positive for mutation, WT=wild type/no amplification/benign/negative for mutation, NR=not reported/not tested

Supplementary Table 6 – Cell markers and RNA signatures		
HUGO symbols	Cell population or score	Type
BLK	B-cells	Cell type
CD19	B-cells	Cell type
MS4A1	B-cells	Cell type
TNFRSF17	B-cells	Cell type
PTPRC	CD45	Cell type
CD8A	CD8 T cells	Cell type
CD8B	CD8 T cells	Cell type
CTSW	Cytotoxic cells	Cell type
GNLY	Cytotoxic cells	Cell type
GZMA	Cytotoxic cells	Cell type
GZMB	Cytotoxic cells	Cell type
GZMH	Cytotoxic cells	Cell type
KLRB1	Cytotoxic cells	Cell type
KLRD1	Cytotoxic cells	Cell type
KLRK1	Cytotoxic cells	Cell type
PRF1	Cytotoxic cells	Cell type
CCL13	Dendritic cells	Cell type
CD209	Dendritic cells	Cell type
HSD11B1	Dendritic cells	Cell type
CD244	Exhausted CD8	Cell type
EOMES	Exhausted CD8	Cell type
LAG3	Exhausted CD8	Cell type
CD163	Macrophages	Cell type
CD68	Macrophages	Cell type
CD84	Macrophages	Cell type
MS4A2	Mast cells	Cell type
TPSAB1/B2	Mast cells	Cell type
CSF3R	Neutrophils	Cell type
FCGR3A/B	Neutrophils	Cell type
S100A12	Neutrophils	Cell type
IL21R	NK CD56dim cells	Cell type
KIR2DL3	NK CD56dim cells	Cell type
KIR3DL1	NK CD56dim cells	Cell type
KIR3DL2	NK CD56dim cells	Cell type
NCR1	NK cells	Cell type
CD3D	T-cells	Cell type
CD3E	T-cells	Cell type

CD3G	T-cells	Cell type
CD6	T-cells	Cell type
SH2D1A	T-cells	Cell type
TBX21	Th1 cells	Cell type
FOXP3	Treg	Cell type
CCL2	Chemokine	Score
CCL4	Chemokine	Score
CCL5	Chemokine	Score
CCL8	Chemokine	Score
CCL18	Chemokine	Score
CCL19	Chemokine	Score
CCL21	Chemokine	Score
CXCL9	Chemokine	Score
CXCL10	Chemokine	Score
CXCL11	Chemokine	Score
CXCL13	Chemokine	Score
GZMA	Cytolytic	Score
PRF1	Cytolytic	Score
CD70	T cell activation	Functional orientation
CD244	T cell activation	Functional orientation
CD48	T cell activation	Functional orientation
CD44	T cell activation	Functional orientation
LAG3	T cell inhibition	Functional orientation
TNFRSF8	T cell inhibition	Functional orientation
CTLA4	T cell inhibition	Functional orientation
TIGIT	T cell inhibition	Functional orientation
PDCD1	T cell inhibition	Functional orientation
HAVCR2	T cell inhibition	Functional orientation
BTLA	T cell inhibition	Functional orientation
ADORA2A	T cell inhibition	Functional orientation
TNFRSF25	T cell inhibition	Functional orientation
LAIR1	T cell inhibition	Functional orientation
FOXP3	Regulatory T cells	Functional orientation
TNFRSF18	Regulatory T cells	Functional orientation
HLA-A	Class I MHC	Functional orientation
HLA-B	Class I MHC	Functional orientation
HLA-C	Class I MHC	Functional orientation
HLA-E	Class I MHC	Functional orientation
HLA-F	Class I MHC	Functional orientation

B2M	Class I MHC	Functional orientation
CCL2	Myeloid cell chemotaxis	Functional orientation
CCL5	Myeloid cell chemotaxis	Functional orientation
VEGFA	Myeloid cell chemotaxis	Functional orientation
CSF1	Myeloid cell chemotaxis	Functional orientation
CCL2	M1 markers	Functional orientation
CXCL10	M1 markers	Functional orientation
GBP2	M1 markers	Functional orientation
IFIT3	M1 markers	Functional orientation
SLAMF7	M1 markers	Functional orientation
CXCL9	M1 markers	Functional orientation
CCL8	M1 markers	Functional orientation
IL1B	M1 markers	Functional orientation
CD38	M1 markers	Functional orientation
CXCL16	M2 markers	Functional orientation
CXCR4	M2 markers	Functional orientation
CD14	M2 markers	Functional orientation
MRC1	M2 markers	Functional orientation
ARG1	M2 markers	Functional orientation
CCL13	M2 markers	Functional orientation
MRC1	M2 markers	Functional orientation
CCL2	Tertiary lymphoid structure	Functional orientation
CCL4	Tertiary lymphoid structure	Functional orientation
CCL5	Tertiary lymphoid structure	Functional orientation
CCL8	Tertiary lymphoid structure	Functional orientation
CCL18	Tertiary lymphoid structure	Functional orientation
CCL19	Tertiary lymphoid structure	Functional orientation
CCL21	Tertiary lymphoid structure	Functional orientation
CXCL9	Tertiary lymphoid structure	Functional orientation
CXCL10	Tertiary lymphoid structure	Functional orientation
CXCL11	Tertiary lymphoid structure	Functional orientation

CXCL13	Tertiary lymphoid structure	Functional orientation
VEGFA	Angiogenic markers	Functional orientation
VEGFB	Angiogenic markers	Functional orientation
KDR	Angiogenic markers	Functional orientation
CXCR2	Angiogenic markers	Functional orientation
HIF1A	Angiogenic markers	Functional orientation
ANGPT2	Angiogenic markers	Functional orientation
IDO1	IFNg signaling	Score
CXCL10	IFNg signaling	Score
CXCL9	IFNg signaling	Score
HLA-DRA	IFNg signaling	Score
STAT1	IFNg signaling	Score
IFNG	IFNg signaling	Score
CD3D	IFNg downstream	Score
IDO1	IFNg downstream	Score
CD3E	IFNg downstream	Score
CCL5	IFNg downstream	Score
GZMK	IFNg downstream	Score
CD2	IFNg downstream	Score
HLA-DRA	IFNg downstream	Score
CXCL13	IFNg downstream	Score
IL2RG	IFNg downstream	Score
NKG7	IFNg downstream	Score
HLA-E	IFNg downstream	Score
CXCR6	IFNg downstream	Score
LAG3	IFNg downstream	Score
CXCL10	IFNg downstream	Score
STAT1	IFNg downstream	Score
GZMB	IFNg downstream	Score

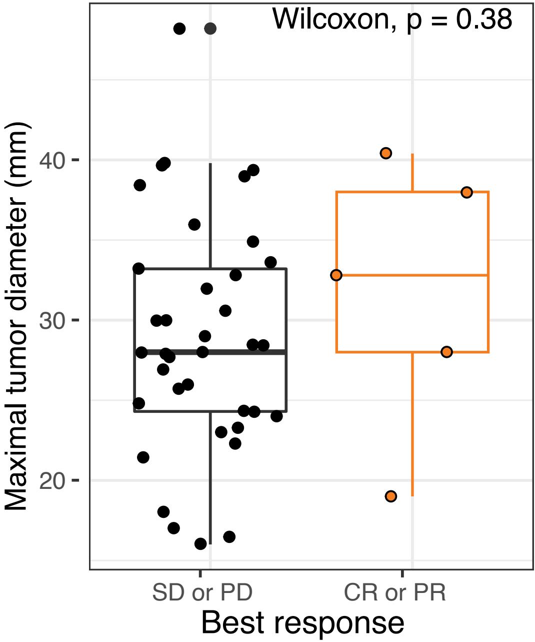

Figure S1. Tumor size comparisons between responders and non-responders in patients who received declared dose DNX-2401 and pembrolizumab (n=42 independent samples). Shown are the distribution of maximal tumor size in mm for each patient. On the left are patients who had either stable disease (SD) or progressive disease (PD), and on the right are patients who had complete response (CR) or partial response (PR). Central bars of boxplots indicate medians, the box defines the upper and lower quartiles of the distribution, and the whiskers define the 1.5× interquartile range (IQR)

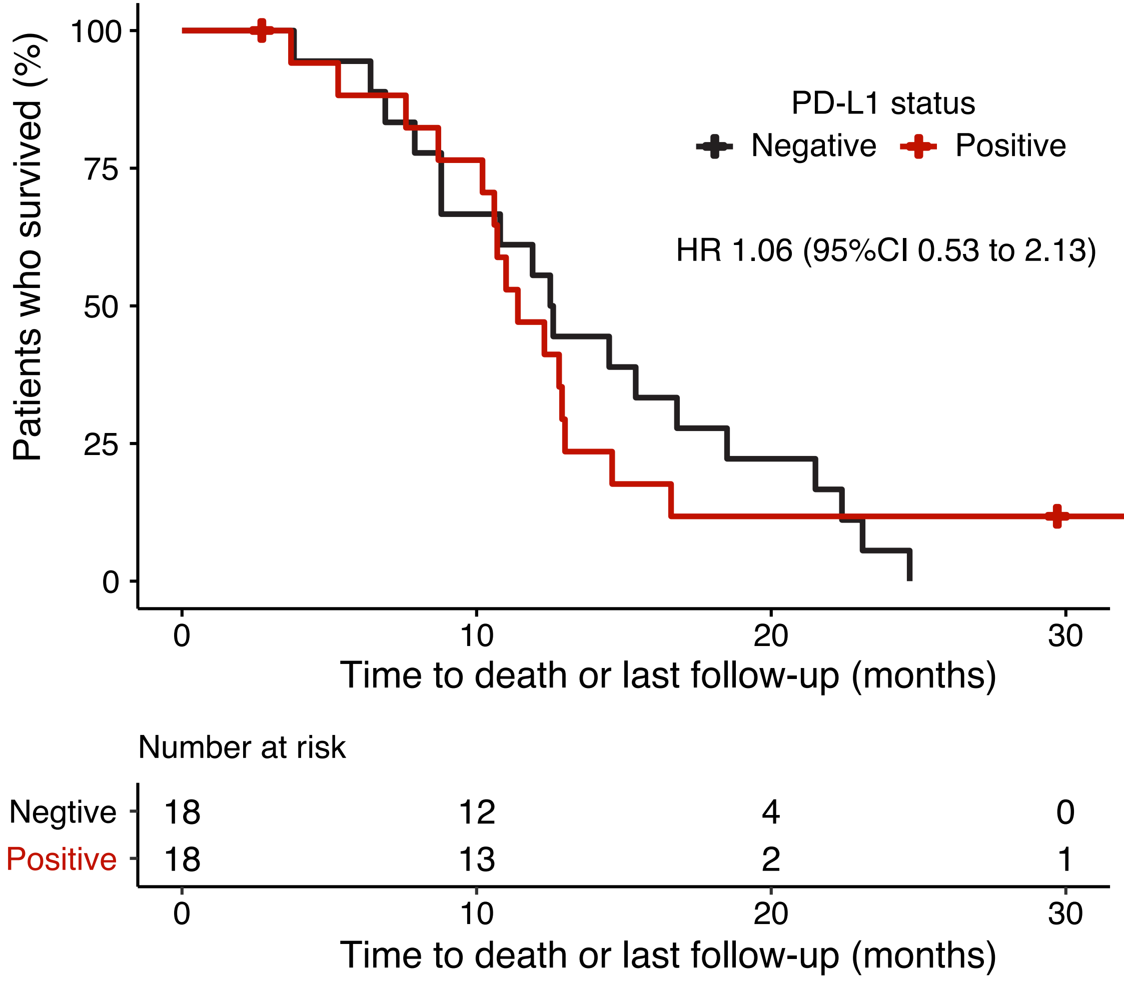

Supplementary Figure 2. Survival of patients treated with $5x10^{10}$ DNX-2401 and pembrolizumab. + denote censored data. Patients stratified according to response to tumor expression of PD-L1 by immunohistochemistry (n=18 positive and n=18 negative).
