# Supplementary material for: Oncolytic DNX-2401 virotherapy plus pembrolizumab in recurrent glioblastoma: a phase 1/2 trial
Source: Nat Med. 2023 May 15;29(6):1370–8. doi: 10.1038/s41591-023-02347-y (PMC10287560; doi:10.1038/s41591-023-02347-y)
Supplement: Supplementary file 2 — Reporting Summary [file 41591_2023_2347_MOESM2_ESM.pdf]

Corresponding author(s): Gelareh Zadeh

Last updated by author(s): 04/03/2023

Reporting Summary

Nature Portfolio wishes to improve the reproducibility of the work that we publish. This form provides structure for consistency and transparency in reporting. For further information on Nature Portfolio policies, see our Editorial Policies and the Editorial Policy Checklist.

Statistics

For all statistical analyses, confirm that the following items are present in the figure legend, table legend, main text, or Methods section.

n/a	Confirmed	
☐	☒	The exact sample size (n) for each experimental group/condition, given as a discrete number and unit of measurement
☐	☒	A statement on whether measurements were taken from distinct samples or whether the same sample was measured repeatedly
☐	☒	The statistical test(s) used AND whether they are one- or two-sided<br>*Only common tests should be described solely by name; describe more complex techniques in the Methods section.*
☐	☒	A description of all covariates tested
☒	☐	A description of any assumptions or corrections, such as tests of normality and adjustment for multiple comparisons
☐	☒	A full description of the statistical parameters including central tendency (e.g. means) or other basic estimates (e.g. regression coefficient) AND variation (e.g. standard deviation) or associated estimates of uncertainty (e.g. confidence intervals)
☐	☒	For null hypothesis testing, the test statistic (e.g. F, t, r) with confidence intervals, effect sizes, degrees of freedom and P value noted<br>*Give P values as exact values whenever suitable.*
☒	☐	For Bayesian analysis, information on the choice of priors and Markov chain Monte Carlo settings
☒	☐	For hierarchical and complex designs, identification of the appropriate level for tests and full reporting of outcomes
☒	☐	Estimates of effect sizes (e.g. Cohen's d, Pearson's r), indicating how they were calculated

Our web collection on statistics for biologists contains articles on many of the points above.

Software and code

Policy information about availability of computer code

Data collection	SAS 9.4
Data analysis	SAS 9.4, R 4.1.3, HALO v3.031, Horos v3.3.6

For manuscripts utilizing custom algorithms or software that are central to the research but not yet described in published literature, software must be made available to editors and reviewers. We strongly encourage code deposition in a community repository (e.g. GitHub). See the Nature Portfolio guidelines for submitting code & software for further information.

Data

Policy information about availability of data

All manuscripts must include a data availability statement. This statement should provide the following information, where applicable:

- Accession codes, unique identifiers, or web links for publicly available datasets
- A description of any restrictions on data availability
- For clinical datasets or third party data, please ensure that the statement adheres to our policy

Pseudononymized participant data, including outcomes and relevant reported patient characteristics, are shared as source data. Processed gene expression data that can be linked to pseudonymized participant data are provided at GSE226976. Previously published data was accessed from SRAPRJNA482620 with clinical annotation provided from authors. Custom algorithms or software were not used to generate the results reported in this manuscript.

Human research participants

Policy information about studies involving human research participants and Sex and Gender in Research.

Reporting on sex and gender	Female (N=20) and male (N=29) patients were included in this study
Population characteristics	49 patients from 13 of the 15 participating institutions were enrolled. The demographic and baseline clinical characteristics of all patients enrolled are reported in Table 1. The median age of patients was 53 years and 41% were women. The majority of patients (80%) presented after first recurrence and 18% of patients were using steroids at baseline. All patients had histopathological diagnosis of glioblastomas, except one patient enrolled with gliosarcoma (2%). Most patients (90%, N=44) had reported IDH1 wildtype tumors, 4 (8%) had IDH1 mutant tumors, and IDH1 mutation status was not known for 1 patient. All patients had received prior treatment with temozolomide and radiotherapy, 6 (12%) patients had prior bevacizumab treatment, and 5 (10%) had prior treatment with a tumor-treating fields device.
Recruitment	From September 2016 to January 2019. Patients were recruited by local investigators. All patients signed consent forms.
Ethics oversight	This study was approved by the University of Arkansas for Medical Sciences IRB, Ohio State University Cancer IRB, University of Utah IRB, MD Anderson Cancer Center Western IRB (central), Weill Cornell Medical College IRB, Cleveland Clinic IRB, Office of the Human Research Protection Program (OHRPP) UCLA Medical IRB, Northwestern University Office for the Protection of Research Subjects IRB, Texas Oncology Western IRB (central), Memorial Sloan Kettering Cancer Center Institutional Review and Privacy Board, Lehigh Valley Health Network IRB/Research Participant, Rutgers HealthSci IRB, UNC at Chapel Hill Office of Human Research Ethics, University of Minnesota Human Research Protection Program, University Health Network Research Ethics Board

Note that full information on the approval of the study protocol must also be provided in the manuscript.

Field-specific reporting

Please select the one below that is the best fit for your research. If you are not sure, read the appropriate sections before making your selection.

☒ Life sciences ☐ Behavioural & social sciences ☐ Ecological, evolutionary & environmental sciences

For a reference copy of the document with all sections, see nature.com/documents/nr-reporting-summary-flat.pdf

Life sciences study design

All studies must disclose on these points even when the disclosure is negative.

Sample size	Assuming a historical control response rate equal to 5%, we estimated that a sample size of 39 evaluable patients would provide the trial with 80% power (at one-sided significance level of 5%) to detect an objective response rate of 18%. Type I error will be set at 5% (one-sided), so the 90% CI will also be provided
Data exclusions	No additional inclusion/exclusion criteria were applied beyond that determined in the trial protocol.
Replication	This is a prospective, single arm, non-randomized phase 1/2 trial. Genomic analyses were performed once per biological patient sample due to availability of tissue sample and expense of sequencing
Randomization	This was a non-randomized single-arm trial. Aggregate endpoints are reported and comparisons were made with historical control rates.
Blinding	The investigators were not blinded per study design and was not placebo controlled. Correlative analyses were conducted blinded to patient outcome using de-identified samples

Reporting for specific materials, systems and methods

We require information from authors about some types of materials, experimental systems and methods used in many studies. Here, indicate whether each material, system or method listed is relevant to your study. If you are not sure if a list item applies to your research, read the appropriate section before selecting a response.

Materials & experimental systems

n/a	Involved in the study
☐	☒ Antibodies
☒	☐ Eukaryotic cell lines
☒	☐ Palaeontology and archaeology
☒	☐ Animals and other organisms
☐	☒ Clinical data
☒	☐ Dual use research of concern

Methods

n/a	Involved in the study
☒	☐ ChIP-seq
☒	☐ Flow cytometry
☒	☐ MRI-based neuroimaging

Antibodies

Antibodies used

Immunohistochemistry
anti-CD3 (Agilent, M725401-2, mouse monoclonal, clone F7.2.38, 1:100)
anti-IBA1 (Wako, 019-19741, rabbit polyclonal, clone Synthetic peptide (Iba1 C-terminal sequence), 1:1500)
anti-CD68 (Agilent, M0514, mouse monoclonal, clone KP1, 1:200),
anti-CD4 (abcam, ab133616, rabbit monoclonal, clone EPR6855, 1:100)
anti-CD8 (abcam, ab93278, rabbit monoclonal, clone EP1150Y, 1:250).
EnVision+ Single Reagent (HRP. Rabbit) Agilent, K4003, goat, labeled polymer
EnVision+ Single Reagent (HRP. Mouse Agilent, K4001, goat, labeled polymer

Immunofluorescence
CD11b (Rabbit monoclonal, clone EPR1344, 1:1000, Abcam, product number ab133357),
CD163 (Mouse monoclonal, clone MRQ-26, ready-to-use, Cell Marque, product number 760-4437),
CD3 (Rabbit polyclonal, IgG, ready-to-use, Agilent, product number IR503),
CD8 (Mouse monoclonal, clone C8/144B, ready-to-use, Agilent, product number IR623),
GFAP (Mouse monoclonal, clone 6F2, 1:500, Agilent, product number M0761)

Validation

For PD-L1: https://www.agilent.com/en-ca/products/pharmdx/pd-l1-ihc-22c3-pharmdx-testing
Validation of antibodies for immunofluoresence has been previously reported PMID 35767439
Validation for Immunohistochemical antibodies:
Iba-1 PMIDs 8713135, 9630473, 10934045, 11500035, 11916959
CD4 PMIDs 32878426, 33674617, 33718516, 33762733
CD8 PMIDs 32929219, 33397441, 34018092, 33425353, 33664865
In house validation for immunohistochemical antibodies:
anti-CD3 human liver at 1:100, anti-CD68 human cortex at 1:200, anti-Iba-1 human cortex 1:1500, anti-CD4 human spleen 1:100, anti-CD8 human spleen 1:100.

Clinical data

Policy information about clinical studies
All manuscripts should comply with the ICMJE guidelines for publication of clinical research and a completed CONSORT checklist must be included with all submissions.

Clinical trial registration

NCT02798406

Study protocol

The study protocol is included with Supplementary material of this manuscript.

Data collection

Recruitment from September 28 2016 to January 17 2019 across 13 institutions (University of, University of Utah, MD Anderson Cancer Center, Weill Cornell Medical College, Cleveland Clinic, University of California Los Angeles, Northwestern University, Texas Oncology, Memorial Sloan Kettering Cancer Center, Rutgers, University of North Carolina, University of Minnesota, University Health Network Research Ethics Board). Data collection until June 2021.

Outcomes

The primary safety objective was to evaluate the safety of escalating doses of DNX-2401 and the overall safety of the declared dose of intratumoral DNX-2401 when followed by sequential intravenous administration of pembrolizumab. Adverse events and serious adverse events were summarized for all patients in the study and were considered treatment-related if reported as possibly, probably, or definitely related to study drug

The primary efficacy objective was to determine the objective response rate, defined as the percentage of patients that had complete or partial responses based on mRANO criteria

Secondary efficacy objectives were to evaluate 12-month overall survival as well as the clinical benefit rate, defined as the proportion of patients treated with DNX-2401 and pembrolizumab who had stable disease, complete response, or partial response. Overall survival was defined as the time from the start of treatment (DNX-2401 injection) until death (or last follow-up). Overall survival at 12 months was summarized using Kaplan-Meier methods and outcomes were compared to historical rates of 20% from an approved treatment approach.
